# TFEB regulates pluripotency transcriptional network in mouse embryonic stem cells independent of autophagy–lysosomal biogenesis

**DOI:** 10.1038/s41419-021-03632-9

**Published:** 2021-04-01

**Authors:** Anderson Tan, Renuka Prasad, Eek-hoon Jho

**Affiliations:** Department of Life Science, University of Seoul, Seoul, 130-743 Republic of Korea

**Keywords:** Embryonic stem cells, Self-renewal

## Abstract

Transcription factor EB (TFEB), a well-known master regulator of autophagy and lysosomal biogenesis, is a member of the microphthalmia family of transcription factors (MiT family). Over the years, TFEB has been shown to have diverse roles in various physiological processes such as clearance for intracellular pathogenic factors and having developmental functions such as dendritic maturation, as well as osteoclast, and endoderm differentiation. However, in the present study, we propose a novel mechanism for TFEB governing pluripotency of mouse ESCs (mESCs) by regulating the pluripotency transcriptional network (PTN) in these cells. We observed high levels of TFEB mRNA and protein levels in undifferentiated mESCs. Interestingly, we found a reduction of Nanog and Sox2 levels in TFEB knockout (KO) mESCs while pluripotency was maintained as there was an upregulation of TFE3, a potent stem cell maintenance factor. In consistent, double knockout of TFEB/TFE3 (TFEB/3 DKO) reduced mESC pluripotency, as indicated by the loss of ESC morphology, reduction of ESC markers, and the emergence of differentiation markers. We further discovered that *Nanog* was a TFEB target gene in undifferentiated mESCs. TFEB also promoted *sex-determining region Y-box2* (*Sox2*) transcription by forming a heterodimer with Sox2 in mESCs. Notably, Sox2, Oct4, and Nanog were also binding to the *TFEB* promoter and thus generating a feed-forward loop in relation to TFEB. Although high levels of nuclear TFEB are expected to enhance autophagy–lysosomal activity, undifferentiated mESC remarkably displayed low basal autophagy–lysosomal activity. Overexpression or knockout of *TFEB* did not affect the expression of TFEB lysosomal–autophagy target genes and TFEB also had a lesser binding affinity to its own lysosomal promoter-target genes in mESCs compared to differentiated cells. Collectively, these findings define a newly incorporative, moonlighting function for TFEB in regulating PTN, independent of its autophagy–lysosomal biogenesis roles.

## Introduction

Stem cells are often characterized by an intrinsic and flexible cellular potential to self-renew and differentiate into various functional cell types. It is generally accepted that the self-renewal and differentiation potential of stem cells requires precise control of protein turnover and lysosome-mediated degradation of organelles^1^. Autophagy, an evolutionarily ancient stress-responsive process, has been recently recognized as one of the major mechanisms through which cells sustain their precise morphology and function via regulating protein turnover, particularly of toxic cytosolic entities^2–6^. Autophagy is also required for pluripotency of cancer stem cells and has also been reported to be necessary for the maintenance of stemness in muscle stem cells^7,8^. For coordinating the autophagy–lysosomal pathway, a finely tuned transcriptional regulation of this pathway enables cells to adapt to various environmental cues^9–11^.

Transcriptional activity of transcription factor EB (TFEB), a member of the microphthalmia family of basic helix–loop–helix–leucine-zipper (bHLH-Zip) transcription factors (MiT family), is salient in controlling autophagy-lysosomal biogenesis and metabolics^12,13^. From a developmental perspective, *TFEB*-knockout (KO) mice have been shown to cause embryonic lethality at E10.5 due to defective placental vascularization^14^. In addition, TFEB depletion has been reported to be associated with defective differentiation into osteoclast and endodermal lineages^15,16^. Recently, it was reported that the transcriptional activity of transcription factor E3 (TFE3), which is the most related to TFEB in the MiT family, is imperative for the maintenance of pluripotency by blocking pluripotency exit^17,18^. Interestingly, in-depth proteomics analysis of mouse embryos during development has also revealed relatively high expression of TFEB^19^. However, a potential role for TFEB in maintaining pluripotency of mESC has not been described.

In the present study, we found that TFEB was highly expressed in mESCs. However, TFEB KO did not impair mESC pluripotency due to TFE3 compensation which blocks pluripotency exit. In agreement, double KO of TFEB and TFE3 impaired mESC self-renewal, as indicated by the loss of ESC colony morphology, reduction of ESC markers, and increase of differentiation markers. Mechanistically, TFEB was found to specifically bind to the *Nanog* promoter in mESCs and to form a heterodimer with sex-determining region Y-box2 (Sox2), thus promoting *Sox2* transcription. As such, *Nanog* and *Sox2* may be considered as newly discovered physiological targets of TFEB. Interestingly, we also found that Sox2, Oct4, and Nanog bound to the *TFEB* promoter. In addition, we found that the regulation of mESCs pluripotency did not require autophagy-lysosomal biogenesis mediated by TFEB. Collectively, our findings suggest that TFEB regulates PTN independently of autophagy–lysosomal biogenesis.

## Results

### Dynamic changes in TFEB levels during mESC differentiation

In western blot and real-time polymerase chain reaction (PCR) analyses, the levels of endogenous TFEB mRNA and protein were observed to be high in undifferentiated cells, and the levels were subsequently reduced during differentiation induced by leukemia inhibitory factor (LIF) withdrawal (Supplementary Fig. 1a, b) or during embryonic body (EB) formation (Supplementary Fig. 1c, d). However, TFEB levels were upregulated from Day 7, supporting the role of TFEB during differentiation that has been previously reported^15,16^. The levels of Oct3/4, which is a stem cell marker, were consistently reduced during differentiation (Supplementary Fig. 1a–d). In addition, differentiation markers from endoderm, ectoderm, and mesoderm lineages were also significantly upregulated during differentiation (Supplementary Fig. 1e, f). These also validate the feasibility and reliability of the differentiation technique that has been employed in the present study.

In addition, we also checked the expressions of MiT family members (TFE3, MITF, and TFEC) to show TFEB specificity in mESC during differentiation (mESC, D5, and D9) (Supplementary Fig. 1g). To examine other genes’ relative expression, Day 5 (D5) sample was selected as a control due to the lowest TFEB expression among three differentiation stages. We found high levels of TFEB and TFE3 in undifferentiated mESC. This is consistent with previous literature where TFE3 is a potent stem cell maintenance factor^17^. TFEB levels were also relatively high in the D9 sample consistent with its role in differentiation^15,16^. However, the relative expression of MITF was low and TFEC was negligible in undifferentiated mESC, which suggests that TFEB and TFE3 are the major MiT family members in the undifferentiated mESCs. High levels of endogenous TFEB observed in undifferentiated cells raised the possibility that TFEB plays as yet unrecognized role in the regulation of pluripotency of mESCs.

### Generation and characterization of TFEB KO and TFEB/3 DKO mESCs

**Fig. 1:**
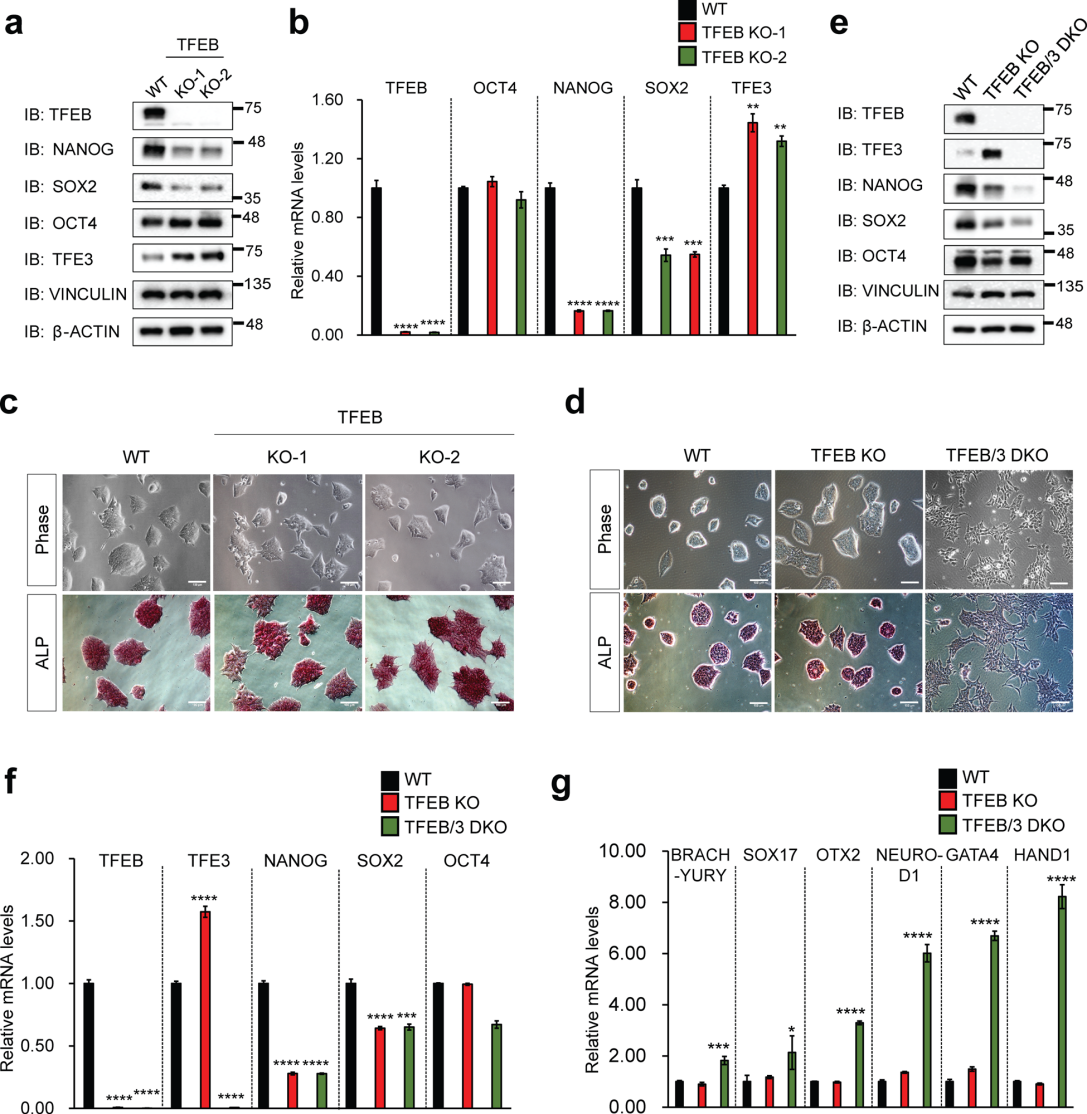
Generation and characterization of TFEB KO mESCs. **a** Lysates of TFEB KO mESCs shown in (**c**) were prepared and western blotting was performed using antibodies as indicated. Protein levels of vinculin and β-actin were used as loading controls. **b** The gene expressions of stemness markers *Oct4*, *Nanog,* and *Sox2* were analyzed through qPCR in TFEB KO mESCs. mRNA was normalized with β-actin. (**c**) After puromycin selection, TFEB KO clones (KO-1 & KO-2) were grown in an mESC culture medium. Morphology (top panels) and alkaline phosphatase (ALP) staining (bottom panels) results of TFEB wildtype (WT) and TFEB KO are shown. Scale bars, 100 μm. **d** WT, TFEB KO, and TFEB/3 DKO cells were grown in an mESC culture medium. Pictures of morphology (top panels) and ALP staining (bottom panels) of WT, TFEB KO, and TFEB/3 DKO mESCs are shown. Scale bars, 100 μm. **e** Lysates of WT, TFEB KO, and TFEB/3 DKO mESCs shown in (**d**) were prepared and western blotting was performed using antibodies as indicated. Protein levels of vinculin and β-actin were used as loading controls. **f** The expression of genes for stemness markers *Oct4*, *Nanog*, and *Sox2* was analyzed through qPCR in WT, TFEB KO, and TFEB/3 DKO mESCs. **g** Expressions of genes for differentiation markers of ectoderm lineage (Otx2, NeuroD1), mesoderm lineage (Brachyury, Hand1), and endoderm lineage (Sox17, Gata4) were analyzed through qPCR in WT, TFEB KO, and TFEB/3 DKO mESCs. All mRNA was normalized with β-actin. All statistical analyses represent average values of a representative experiment from at least two independent experiments. Error bars represent SD values of triplicate assays. Data are shown as mean ± SD, *n* = 3. *p < 0.05; **p < 0.01; ***p < 0.001 ****p < 0.0001 compared to the corresponding control group. The student’s *t* test was used for all statistical analysis.

To elucidate the function of TFEB in mESCs, we generated the TFEB KO mESCs by using CRISPR/Cas9 system. Three independent guide RNAs (gRNAs) targeting different exons of TFEB were introduced into mESCs by lentivirus transduction and we isolated independent clones that showed a complete KO of TFEB (Fig. 1a, b). Initial characterization of the TFEB KO mESCs did not reveal any overt differences compared to wild-type (WT) counterparts. TFEB KO mESCs retained typical ESC-like colony morphology, and similarly, the cells displayed comparable levels of alkaline phosphatase (ALP) staining, an ESC-specific marker (Fig. 1c). However, mRNA and protein levels of ESC core markers, Sox2 and Nanog but not Oct4, were significantly decreased in these cells (Fig. 1a, b). We were puzzled by this outcome and speculated that there might be a functional redundancy driven by TFE3, the closest member to TFEB. TFE3 has been previously reported as a potent stem cell maintenance factor as it blocks pluripotency exit and increases resistance to differentiation^17^. To this end, we generated TFEB/TFE3 double KO mESCs (TFEB/3 DKO) and examined the pluripotency status. We observed a more flattened and widespread morphology of TFEB/3 DKO cells in comparison to the compact colony morphology of WT and TFEB KO cells (Fig. 1d). This is an indication of differentiation occurring in TFEB/3 DKO cells. TFEB/3 DKO cells also displayed significant reduction of ALP staining compared to WT and TFEB KO cells (Fig. 1d). In addition, a significant reduction of mESC stemness markers, Nanog and Sox2, at both mRNA and protein levels in TFEB/3 DKO cells was observed (Fig. 1e, f). Differentiation markers of 3 different germ layers (ectoderm (Otx2, NeuroD1), mesoderm (Brachyury, Hand1) and endoderm (Sox17, Gata4)) were also upregulated in TFEB/3 DKO cells (Fig. 1g). In this regard, we observed TFE3 compensation in TFEB KO mESCs occurring which could explain why TFEB KO did not impair stem cell pluripotency. Along with that, we further checked if the loss of pluripotency in TFEB/3 DKO cells was not due to autophagy/lysosomal inhibition. To achieve that, we knockdown ATG7, a well-known autophagy initiation factor, or inhibit autolysosome formation by treating the cells with Bafilomycin A1. We found that neither ATG7 knockdown nor Bafilomycin A1 treatment affected mESC pluripotency as shown by the unaltered compact colony morphology compared to the control group (Supplementary Fig. 2a). The levels of mESC markers were not largely affected as well (Supplementary Fig. 2b). Overall these results suggest that the phenotype seen in TFEB/3 DKO cells is due to the lack of pluripotency regulatory role of TFEB/3 but not inhibition of autophagy/lysosomal function.

### Nanog is a TFEB target gene in undifferentiated mESCs

**Fig. 2:**
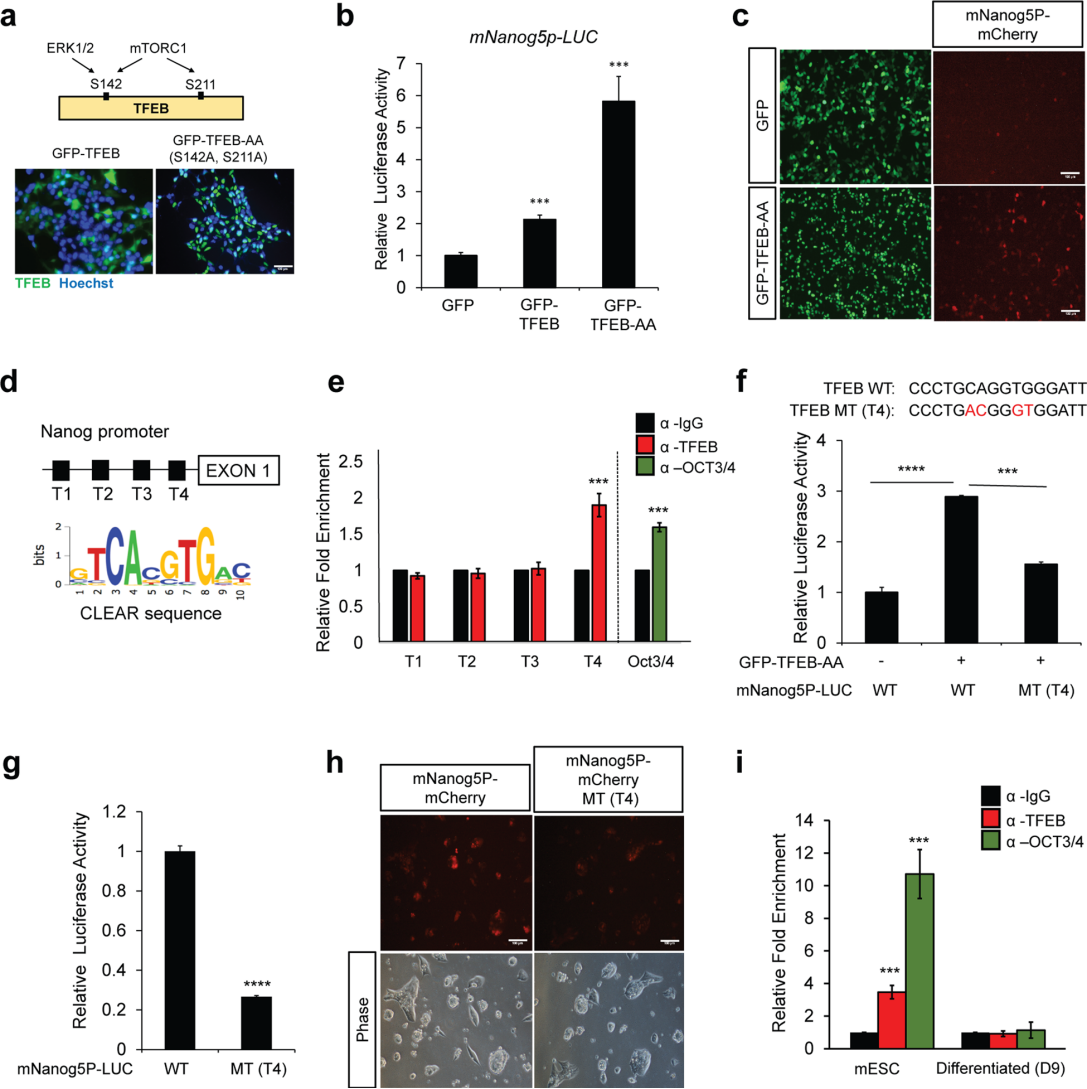
Nanog is a TFEB target gene in undifferentiated mESCs. **a** Construction of constitutive active TFEB (TFEB-AA) by mutating serine 142 (S142) and serine 211 (S211) to alanine (S142A & S211A). TFEB-AA was found mainly in the nucleus of HEK293T cells upon transfection. Scale bars, 100 μm. **b** Overexpression of TFEB was observed to increase *Nanog* promoter driven-luciferase activity (mNanog5p-LUC) in HEK293T cells. **c** *Nanog* promoter driven-mCherry (mNanog5p-mCherry) activity is enhanced by the active form of TFEB (GFP-TFEB-AA) in HEK293T cells. Scale bars, 100 μm. **d** Presence of four putative TFEB binding sites (T1–T4) (CLEAR sequence) at the mouse Nanog promoter site. **e** ChIP-qPCR shows high enrichment of TFEB at the T4 site in undifferentiated mESCs. OCT3/4 pulled down served as a positive control. **f** Introduction of mutation at T4 site (MT (T4)) reduced TFEB-AA-mediated Nanog promoter-driven luciferase activity as compared to wildtype (WT) in HEK293T cells. **g** mESCs transfected with MT(T4) reporter displayed lower Nanog luciferase activity as compared to WT reporter. **h** *Nanog* promoter-driven-mCherry with T4 mutation site (mNanog5p-mCherry MT(T4)) activity is reduced compared to control in mESCs. Scale bars, 100 μm. **i** ChIP-qPCR shows high enrichment of TFEB at T4 site in undifferentiated mESCs, but not in Day 9 differentiated EB (D9). All statistical analyses represent average values of a representative experiment from at least two independent experiments. Error bars represent SD values of triplicate assays. Data are shown as mean ± SD, *n* = 3. *p < 0.05; **p < 0.01; ***p < 0.001 ****p < 0.0001 compared to the corresponding control group. The student’s *t* test was used for all statistical analysis.

Sox2, Oct4, and Nanog form the pluripotency transcriptional network (PTN) that intricately governs pluripotency and cell fate determination^20^. As we observed a reduction of Nanog and Sox2 levels in TFEB KO cells (Fig. 1), we further sought to gauge if Nanog and Sox2 were physiological targets of TFEB in mESCs. TFEB subcellular localization is mainly regulated by phosphorylation of its specific serine residues and mutation of these serine residues 142/211 (S142/S211) into alanine (S142A/S211A) results in the nuclear localization of TFEB and induced expression of target genes^4,21^. To test whether *Nanog* was a potential target gene of TFEB, a constitutively active form of TFEB (TFEB-AA, mutated S142/S211 to S142A/S211A) and a reporter plasmid with a 2.5 kb *Nanog* promoter were generated and transfected in HEK293T cells. TFEB-AA was found to be mainly localized in the nucleus (Fig. 2a). TFEB overexpression was observed to increase the *Nanog*-promoter-driven luciferase activity, and TFEB-AA further enhanced the luciferase activity in these cells (Fig. 2b). TFEB-AA overexpression also enhanced the mCherry positive signal driven by the *Nanog* promoter (Fig. 2c).

TFEB has previously been shown to directly bind to coordinated lysosomal expression and regulation (CLEAR) elements, and promote the expression of the entire set of genes having the CLEAR regulatory motifs in their promoters^3,22^. Interestingly, screening of the *Nanog* promoter uncovered four potentially putative TFEB-binding sites (Fig. 2d). Chromatin immunoprecipitation (ChIP) analysis showed high enrichment of TFEB at the T4 site on the *Nanog* promoter, suggesting that TFEB may bind to this region. ChIP for Oct3/4 was used as a positive control with Oct4 binding to the *Nanog* promoter^23,24^ (Fig. 2e). A substantially reduced reporter activity was also observed after mutation of the T4 site in either HEK293T cells or mESCs (Fig. 2f, g). In addition, a reduction in the mCherry signal driven by the *Nanog* promoter was seen with the T4 site mutation in mESCs (mNanog5P-mCherry MT (T4)) (Fig. 2h). Interestingly, TFEB specifically bound to the *Nanog* promoter in undifferentiated mESCs but not in Day 9 differentiated cells, although the levels of TFEB were observed to be high in Day 9 differentiated cells (Fig. 2i and Supplementary Fig. 1). In addition, we also found that Nanog is regulated only by TFEB but not TFE3. We found that only TFEB overexpression but not TFE3 significantly enhanced Nanog promoter-luciferase activity (Supplementary Fig. 3a). We also performed an endogenous ChIP-qPCR by pulling down TFEB and TFE3 and performing qPCR targeting Nanog promoter. High enrichment of TFEB but not TFE3 was found in Nanog promoter (Supplementary Fig. 3b). Besides that TFE3 knockdown does not affect *Nanog* and *Sox2* mRNA levels (Supplementary Fig. 3c). This data is consistent with previous literature that TFE3 does not bind to Nanog promoter^17^. Taken together, our data suggest that TFEB binds to the promoter of *Nanog* only in the undifferentiated mESC stage.

### TFEB interacts with Sox2 and enhances its expression

We next switched our focus to Sox2, a member of the HMG-box (SOX) family of transcription factors^25,26^. As part of PTN, Sox2 along with Oct4, and Nanog constitute a transcriptional circuitry with a feed-forward regulation, inducing their own expression^27,28^. This type of autoregulation is thought to enhance the stability of the relevant gene expression^29^, contributing to the maintenance of the pluripotent state.

**Fig. 3:**
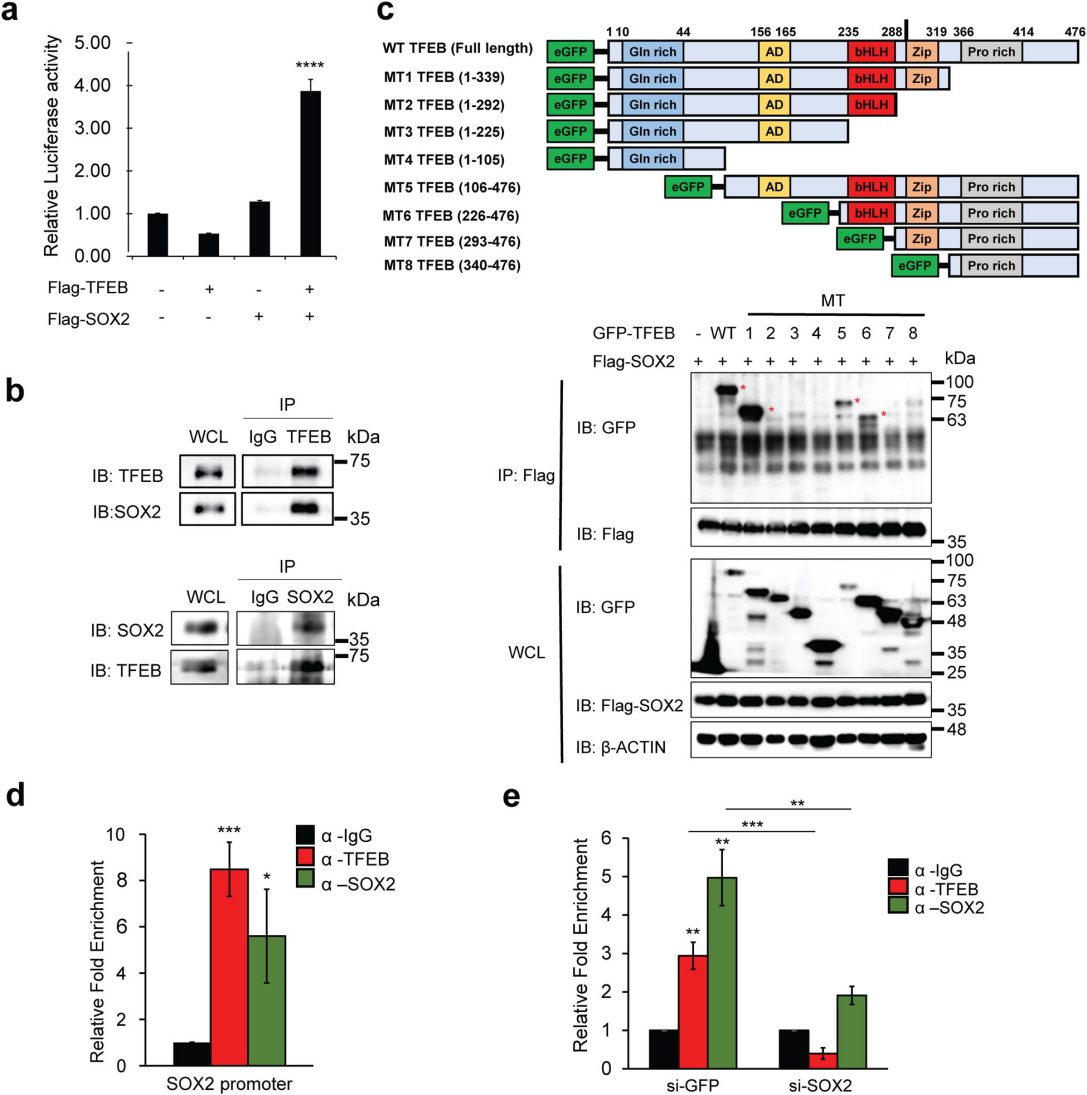
TFEB interacts with Sox2 to enhance the expression of *Sox2*. **a** Co-expression of TFEB and Sox2 significantly enhanced the *Sox2* promoter-driven luciferase activity in HEK293T cells. **b** TFEB interacts with Sox2 at the endogenous level. Immunoprecipitates using mESC lysates were immunoblotted using the indicated antibodies. **c** HEK293T cells were co-transfected with Flag-Sox2, wildtype (WT) TFEB, and various truncated mutant forms of TFEB (MT1-MT8) as indicated in the figure. Abbreviations of each domain are as followed: glutamine-rich (Gln rich), activation domain (AD), basic helix–loop–helix (bHLH), leucine zipper (Zip), and proline-rich (Pro rich). Asterisks (*) indicate interaction. bHLH and Zip domains of TFEB interact with Sox2. **d** ChIP-qPCR analysis was performed by pulling-down with anti-TFEB antibody and anti-Sox2 antibody, following which qPCR targeting the Sox2 binding site at the genomic DNA level in mESCs was performed. **e** Sox2 was knockdown before harvested for ChIP-qPCR analysis. Anti-TFEB antibody and anti-Sox2 antibody were used for pull-down, following which qPCR targeting the Sox2-binding site at the genomic DNA level in mESCs was performed. All statistical analyses represent average values of a representative experiment from at least two independent experiments. Error bars represent SD values of triplicate assays. Data are shown as mean ± SD, *n* = 3. *p < 0.05; **p < 0.01; ***p < 0.001 ****p < 0.0001 compared to the corresponding control group. The student’s *t* test was used for all statistical analysis.

We found that neither Sox2 nor TFEB overexpression could independently increase *Sox2*-promoter-driven luciferase activity. However, co-transfection of *Sox2* and *TFEB* significantly enhanced the luciferase activity of the reporter in HEK 293T cells (Fig. 3a). Sox2 is known to form a heterodimer with Oct4 to activate *Nanog* and *Oct4* genes^23,30^. Therefore, we examined whether Sox2 required TFEB as its interaction partner to promote its own transcription. Co-immunoprecipitation analysis using the mESCs lysates suggested that TFEB endogenously binds to Sox2 (Fig. 3b). Co-immunoprecipitation assays using truncation mutant constructs of TFEB (MT1-MT8 TFEB) showed that only MT1, MT5, and MT6 TFEB interacted with Sox2, suggesting that basic helix–loop–helix (bHLH) and leucine zipper (Zip) domains of TFEB were important for the interaction of TFEB with Sox2 (Fig. 3c). Next, a scan of the Sox2 promoter uncovered the Sox2 binding site. Endogenous ChIP-qPCR by pulling-down TFEB and performing a qPCR detection targeting the Sox2-binding site at the genomic DNA level showed high enrichment of TFEB and Sox2 at the same site (Fig. 3d). In addition, knockdown of Sox2 also significantly reduces TFEB enrichment at the Sox2 promoter (Fig. 3e). Overall, these data suggested that TFEB forms a complex with Sox2 and stimulates the expression of *Sox2*.

In addition, we tested whether TFEB could regulate the expression of Oct4. However, active TFEB overexpression did not have any effect on Oct4-promoter-driven luciferase activity in HEK 293T cells (Supplementary Fig. 4a). Consistently, when Oct4 promoter-GFP and mCherry-TFEB plasmids were co-transfected into HEK 293T cells, no GFP positive signals were detected (Supplementary Fig. 4b). In addition, no interactions were detected between TFEB and Oct4 (Supplementary Fig. 4c). These data suggest that, unlike Sox2, Oct4 is not a physiological target of TFEB.

### TFEB does not induce the expression of genes involved in autophagy and lysosomal biogenesis in undifferentiated mESCs

**Fig. 4:**
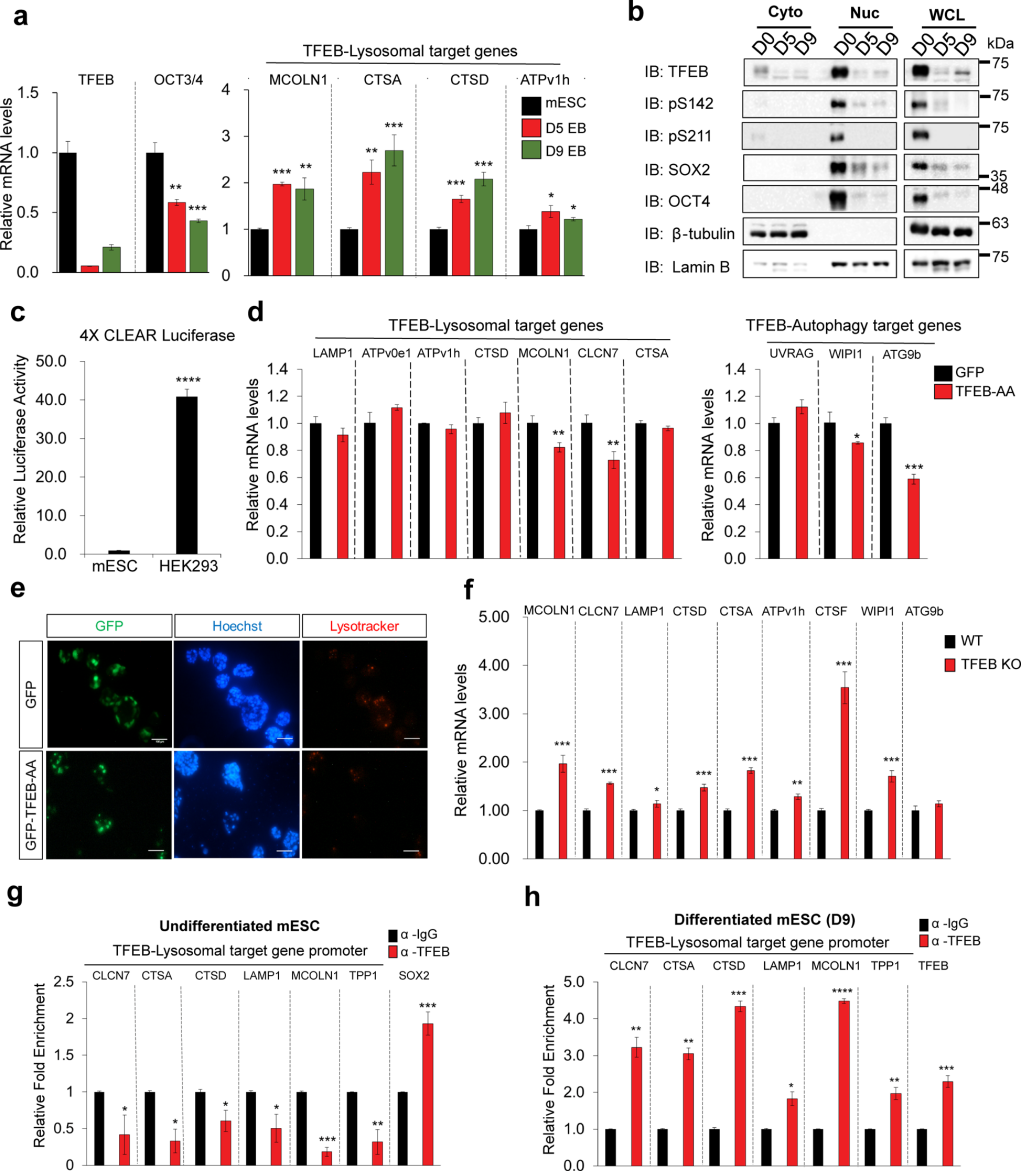
TFEB does not induce expression of genes involved in autophagy and lysosomal biogenesis in undifferentiated mESCs. **a** mRNA expressions of TFEB and TFEB-lysosomal target genes at respective days of embryonic differentiation (EB) (mESC, D5 EB, and D9 EB). **b** Cellular localization of TFEB during EB differentiation (D0, D5, and D9) was determined using cellular fractionation. The phosphorylated form of TFEB (pS142 and pS211) was also analyzed. Sox2 and Oct4 were used as positive controls consistent with their roles as regulators for stemness. β-tubulin and Lamin B were used as cytosol and nuclear loading control, respectively. **c** mESCs and HEK293 cells were transfected with 4XCLEAR reporter for 48 h prior to luciferase analysis. **d** qPCR analysis to measure the expression of TFEB target genes involved in lysosomal biogenesis and autophagy by GFP-TFEB-AA overexpression in mESC. **e** GFP-TFEB-AA was overexpressed in mESC for 48 h prior to labeling with Lysotracker Red. Nuclei were stained with Hoechst. Scale bars, 100 μm. **f** qPCR analysis to measure the expression of TFEB target genes involved in lysosomal biogenesis and autophagy in TFEB KO mESCs. **g**, **h** ChIP-qPCR analysis was performed by pulling-down with anti-TFEB antibody, following which qPCR was performed targeting TFEB-lysosomal target gene promoter binding sites at the genomic DNA level in undifferentiated (**g**) and Day 9 (D9) differentiated (**h**) mESCs. mRNA was normalized with β-actin. All statistical analyses represent average values of a representative experiment from at least two independent experiments. Error bars represent SD values of triplicate assays. Data are shown as mean ± SD, *n* = 3. *p < 0.05; **p < 0.01; ***p < 0.001 ****p < 0.0001 compared to the corresponding control group. The student’s *t* test was used for all statistical analysis.

With TFEB highly expressed in undifferentiated mESCs, we examined whether the lysosomal activity was also high in undifferentiated mESCs. To measure basal lysosomal activity in mESCs, we checked the expression of endogenous TFEB-target genes involved in lysosomal biogenesis during EB differentiation at Days 0, 5, and 9 (Fig. 4a). The expression of genes involved in lysosomal biogenesis was low in undifferentiated mESCs and increased with differentiation. (Fig. 4a). We further strengthen this claim by performing lysotracker staining to check the basal lysosomal activity during EB differentiation (Day 0, Day 5 EB, and Day 9 EB). In accordance with Fig. 4a, we observed a significant increase of lysosomal activity during EB differentiation (Supplementary Fig. 5a). As to monitor autophagy activity (flux), we checked the endogenous LC3 levels during mESC differentiation. As expected, a gradual increase of LC3-2 levels was observed during mESC differentiation (Supplementary Fig. 5b). Even though TFEB is highly expressed in mESC, the level of LC3 is still very low presumably due to its autophagy/lysosomal-independent role in mESC as we claimed in this manuscript. In contrast, a relatively low amount of TFEB in D9 differentiated cells compared to undifferentiated mESC was able to activate autophagy suggesting a context-dependent role of TFEB. However, a concern was that D5 differentiated cells showed high autophagy–lysosomal activity while the level of TFEB is extremely low. We do not know the exact mechanisms, but the autophagy–lysosomal activity may be regulated by TFE3 that is expressed relatively higher than TFEB in D5 mESCs (Supplementary Fig. 1g). This observation pointed to the possibility of TFEB having a lysosomal biogenesis-independent role in mESCs.

Next, we performed a cellular fractionation assay to examine TFEB activity between undifferentiated and differentiated mESCs (Fig. 4b). Although we could not directly compare the levels of TFEB in the cytoplasm and nuclear fraction, TFEB was highly expressed as a similar pattern as positive controls such as Sox2 and Oct4 in undifferentiated mESCs (D0). Lack of β-tubulin in nuclear fraction also shows that TFEB exists in the nucleus of undifferentiated mESCs. Since the total levels of TFEB were low in Day 5 or Day 9 differentiated mESCs, it was not obvious but a small amount of nuclear TFEB was also detected in differentiated cells. Interestingly, phospho-TFEB (S142 and S211), which is normally retained in the cytoplasm, was detected in the nuclear fraction of D0 mESCs. We speculate that there might be unknown mechanisms that permit the entry of TFEB into the nucleus independent of its phosphorylation status.

Under nutrient-rich conditions, undifferentiated mESCs displayed a negligible lysotracker signal; however, lysosomal activity was significantly increased with starvation (Supplementary Fig. 5c). We next used the DQ-BSA assay as a measure of active lysosomal activity. Under basal conditions, the fluorogenic signal of DQ-BSA is low, but once DQ-BSA is digested by intracellular proteases found in lysosomes, individual peptides are released, leading to increased fluorescence. In mESCs, DQ-BSA signals were low under nutrient-rich conditions, and these signals were significantly enhanced with starvation (Supplementary Fig. 5d). In mESCs, starvation-induced lysosomal activity; however, endogenous TFEB levels were reduced (Supplementary Fig. 5e).

Under normal circumstances, TFEB overexpression results in an increase in the number of lysosomes and a higher increment of lysosomal enzymes, thus enhancing the lysosomal catabolic activity^4,22^. Consistently, TFEB overexpression significantly enhanced lysotracker signals and expressions of TFEB target genes involved in lysosomal biogenesis and autophagy in HEK293 cells (Supplementary Fig. 6a–c). Next, we compared the endogenous CLEAR activity in mESCs and HEK293 cells by transfecting respective cells with the 4X-CLEAR luciferase reporter construct (having four copies of the CLEAR element in tandem). Compared to HEK293 cells, mESCs had much lower levels of CLEAR activity despite their relatively high nuclear TFEB expression (Fig. 4c). Interestingly, overexpression of active TFEB (TFEB-AA) in mESCs did not enhance the expression of genes involved in autophagy and lysosomal biogenesis, and some were actually downregulated (*MCOLN1*, *CLCN7*, *WIPI1*, and *ATG9b*) (Fig. 4d). In agreement with this finding, overexpression TFEB-AA in mESCs also did not show any enhancement of lysotracker staining (Fig. 4e). Intriguingly, we also found that TFEB KO partially upregulated autophagy–lysosomal-related TFEB target genes, which is contrary to the observation in other cell types (Fig. 4f). We wondered whether this increase of autophagy and lysosomal group of genes in TFEB KO mESCs was also due to the increased levels of TFE3 (as seen in Fig. 1a, e, f). As expected, TFE3 overexpression increased lysosomal genes expression in mESCs (Supplementary Fig. 7a). Endogenous TFE3 binding in lysosomal promoters in mESC was also validated by ChIP-qPCR (Supplementary Fig. 7b). In addition, we observed increased expression of genes involved in autophagy/lysosomal biogenesis in TFEB KO cells, and this induction was abrogated in TFEB/3 DKO cells (Supplementary Fig. 7c). In consistent, we also observed a significant reduction of lysosomal activity as shown by lysotracker staining in TFEB/3 DKO cells (Supplementary Fig. 7d). Autophagy flux was also increased in TFEB KO cells as shown by the accumulation of LC3-2 and this increase was abolished in TFEB/3 DKO cells (Supplementary Fig. 7e). Overall, our data suggest that the increased autophagy/lysosomal functions in TFEB KO mESC are mediated by TFE3.

To further validate our hypothesis of context-dependent TFEB-autophagy lysosomal target genes, we performed an LC3 flux assay using Bafilomycin A1 in WT and TFEB KO mESCs. We checked LC3 levels in both undifferentiated mESC (WT vs. TFEB KO) and differentiated cells D9 (WT vs. TFEB KO) upon Bafilomycin treatment. We found that LC3-2 levels were slightly increased in Bafilomycin treated-TFEB KO compared to WT mESC. We postulated that this upregulation may be due to the increased of TFE3 levels in TFEB KO cells (Supplementary Fig. 7f). However, LC3-2 levels were significantly reduced in Bafilomycin treated-D9 TFEB KO cells, suggesting that TFEB regulates the expression of genes involved in autophagy–lysosomal biogenesis in D9 differentiated mESC but not in undifferentiated mESCs (Supplementary Fig. 7f). In addition, our ChIP-qPCR assay showed that TFEB had less affinity to its own lysosomal target gene promoters in undifferentiated mESCs compared to Day 9 differentiated mESCs (Fig. 4g, h). Taken together, our compelling evidence suggests that TFEB may have less autophagy/lysosomal biogenesis functions in undifferentiated mESCs.

### TFEB is a downstream target of Sox2, Oct4, and Nanog

**Fig. 5:**
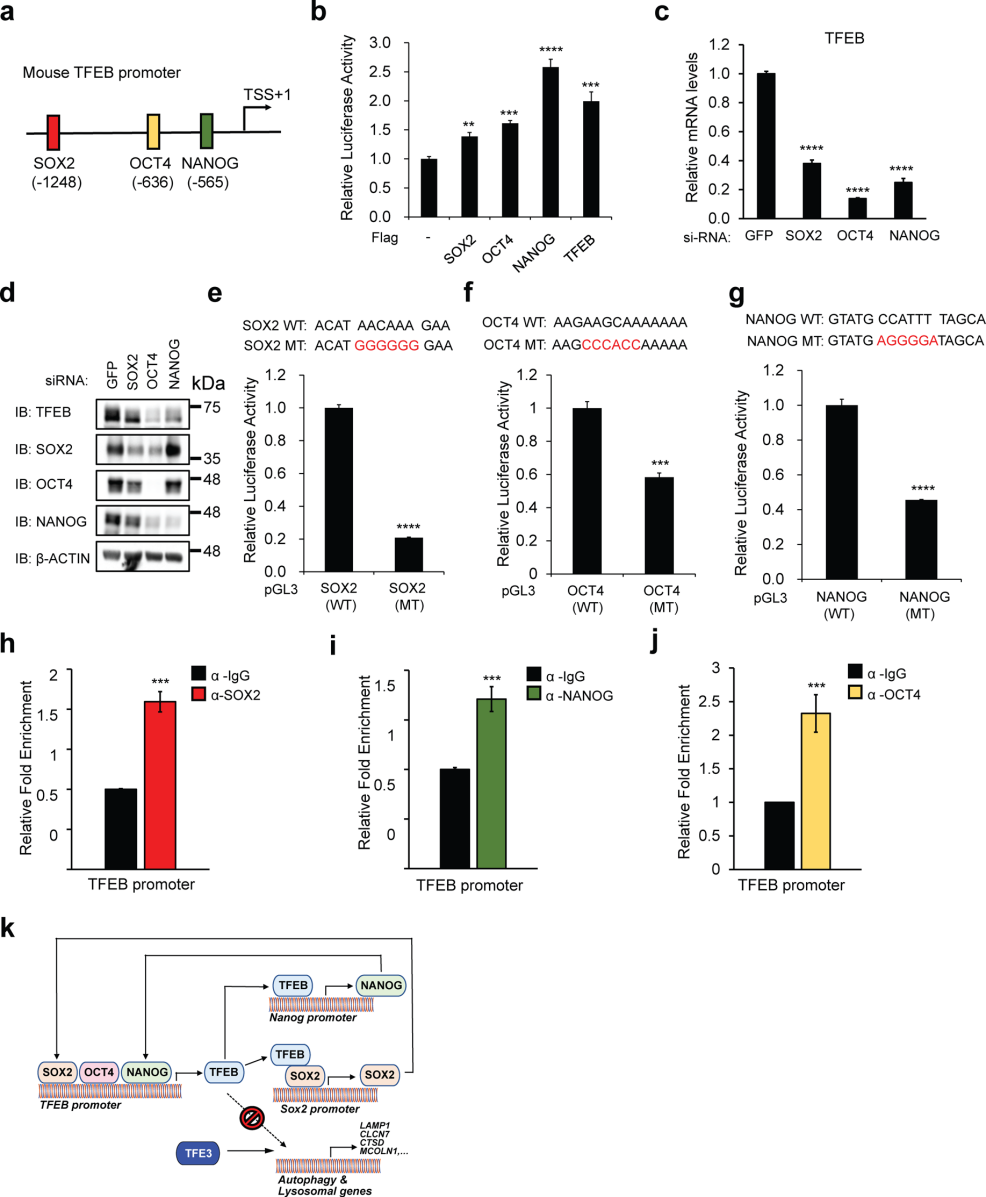
TFEB is a downstream target of Sox2, Oct4, and Nanog. **a** Putative binding sites of Oct3/4, Sox2, and Nanog at mouse TFEB promoter site. **b** Overexpression of Sox2, Oct4, and Nanog increases TFEB promoter-driven luciferase activity in HEK293T cells. **c**, **d** Knockdown of Sox2, Oct3/4, and Nanog in undifferentiated mESCs reduces endogenous TFEB mRNA (**c**) and TFEB protein levels (**d**). **e**–**g** Mutation in putative Sox2, Oct4, or Nanog binding sites (Sox2 MT/Oct4 MT/Nanog MT) in TFEB promoter reduces the TFEB promoter-driven luciferase activity in mESCs. Luciferase activity was evaluated 24 h after transfection. **h**–**j** Endogenous binding of the promoter sequences for Sox2, Oct4, or Nanog at the putative binding sites in TFEB promoter in mESCs was evaluated through ChIP-qPCR analysis. mRNA was normalized with β-actin. **k** Schematic diagram of TFEB in the regulation of pluripotency transcriptional network (PTN) in undifferentiated mESCs. All statistical analyses represent average values of a representative experiment from at least two independent experiments. Error bars represent SD values of triplicate assays. Data are shown as mean ± SD, *n* = 3. *p < 0.05; **p < 0.01; ***p < 0.001 ****p < 0.0001 compared to the corresponding control group. The student’s *t* test was used for all statistical analysis.

Since Sox2, Oct4, and Nanog coordinately occupy the promoters of a host of genes and to maintain the transcriptional program required for pluripotency^31^, we speculated that TFEB may also be a downstream target of these pluripotency proteins in undifferentiated mESC. To determine whether TFEB is a direct target of Sox2, Oct4, and Nanog, we analyzed the promoter of TFEB and identified putative binding sites of these core stem cell markers (Fig. 5a). We found that TFEB-promoter-driven luciferase activity was enhanced by the ectopic expression of Sox2, Oct4, and Nanog (Fig. 5b). TFEB overexpression was also used as a positive control as TFEB transcription is induced through an autoregulatory feedback loop^15^. As a corollary, knockdown of Sox2, Oct4, or Nanog in undifferentiated mESCs also resulted in a reduction of TFEB mRNA levels (Fig. 5c) as well as TFEB protein levels (Fig. 5d). Further experiments testing various mouse TFEB promoter reporters altered with respect to the putative binding sites for Sox2, Oct4, and Nanog in mESCs have been performed. In mESCs, there were significant reductions in luciferase activity of the reporters bearing putative binding site mutations for Sox2, Oct3/4 and Nanog (Sox2(MT), Oct4(MT) and Nanog(MT), respectively) compared to wild-type (WT) mouse TFEB promoter-reporter at endogenous levels (Fig. 5e–g). Ectopic overexpression of recombinant Sox2, Oct4, and Nanog also activated the responding promoters, and mutation of each putative site reduced this activation in HEK 293T cells (Supplementary Fig. 8a–c). To test whether the TFEB gene promoter is a direct target of Sox2, Oct4, and Nanog binding, ChIP-qPCR analysis was performed. Our ChIP-qPCR data showed that Sox2, Oct4, and Nanog bind to the TFEB promoter further substantiated our hypothesis (Fig. 5h–j). Together, these results strongly suggest that Sox2, Oct4, and Nanog directly control TFEB gene expression by binding to the TFEB promoter.

## Discussion

Understanding the molecular basis of ESC pluripotency in the context of stem cell biology research continues to garner intense interest. From what we know, transcriptional regulation is a key component of stem cell maintenance and reprogramming^32–35^. In the present study, we suggest autophagy–lysosomal independent role for TFEB in the regulation of PTN and provide some interesting findings (Fig. 5k). First, the endogenous TFEB mRNA and protein levels were observed to be unexpectedly high in undifferentiated mESCs. This is consistent with the finding that TFEB is strongly expressed during the blastocyst stage of mouse development^19^, although the relevance of TFEB in maintaining stem cell pluripotency with respect to TFEB-related lysosomal biogenesis was not clear. Second, complete KO of TFEB by using the CRISPR/Cas9 system did not impair stem cell pluripotency as there was compensation by the related TFEB family member, TFE3, retarding the pluripotency exit. This was experimentally proven in TFEB/3 DKO cells. Third, we showed a direct link between TFEB and PTN, defining a newly incorporative, moonlighting function for TFEB. PTN consists of a number of master transcription factors, including Sox2, Oct4, and Nanog, which dictate and specify the molecular and transcriptional identity of undifferentiated ESCs^20^. We found that TFEB only binds to the *Nanog* promoter at the undifferentiated stage of mESCs, and in parallel, TFEB also forms a heterodimer with Sox2, thus stimulating *Sox2* transcription. These findings suggest that *Nanog* and *Sox2* are novel physiological targets of TFEB. Fourth, Sox2, Oct4, and Nanog bind to the *TFEB* promoter and enhance the expression of TFEB, generating a feed-forward loop. This may provide a plausible explanation for the high expression of TFEB in undifferentiated mESCs. This is the first study to report a direct relationship between TFEB and the core stem cell factors within the PTN. Finally, we found that undifferentiated mESCs with high levels of nuclear TFEB displayed low basal autophagy flux and lysosomal activity. Traditionally, high expression of TFEB is often associated with the promotion of autophagy and lysosomal biogenesis; however, this was not observed in undifferentiated mESCs. Ectopic expression of TFEB-AA, a nuclear-localized active form of TFEB, also did not induce TFEB-led autophagy and lysosomal target genes (Fig. 4d). Interestingly, KO of TFEB in undifferentiated mESCs also induced TFEB-autophagy and lysosomal target genes (Fig. 4f). We further prove that the increased autophagy–lysosomal functions in TFEB KO mESC are mediated by TFE3 (Supplementary Fig. 7c, d). Differentiated mESCs have lower levels of TFEB than undifferentiated mESCs. However, it may be sufficient to induce TFEB-led autophagy and lysosomal target genes if made accessible and possibly due to epigenetic modifications. This possibility needs to be further examined in the future.

The relationship between the role of TFEB in PTN and traditional autophagy-lysosomal biogenesis is rather interesting but complex. It remains unclear why nuclear TFEB does not upregulate autophagy–lysosomal target genes in undifferentiated mESCs compared to other cell types. It is conceivable that TFEB behaves differently in these cells, possibly due to post-translational modifications that prevent it from binding to the target lysosomal genes. Since TFEB, a member of the MiT family, is a basic bHLH-Zip containing transcription factor, it has a tendency in forming heterodimers with various partners. Our present data suggest that TFEB binds to Sox2 in undifferentiated mESCs. Although it is still speculative at this juncture, we postulate that TFEB target gene expression may vary in a manner that depends on its physiological interactions with other permissible binding partners in various cell types. In addition, interestingly, in Day 9 differentiated mESCs, TFEB did not bind to the *Nanog* promoter (Fig. 2i). This may be due to the differential PTN link with TFEB or an epigenetic modification of Nanog promoter in undifferentiated vs. Day 9 differentiated mESCs. Testing the above-mentioned possibilities may provide new insights into the behavior of TFEB in specialized cell types, such as mESCs, compared to other cell types and the various scenarios for TFEB function.

## Materials and methods

### Cell lines and cell culture methods

HEK293T and HEK293 cell lines were maintained in high glucose Dulbecco’s Modified Eagle Medium, supplemented with 10% fetal bovine serum (FBS), 0.1 mM MEM nonessential amino acids (NEAA), 2 mM l-glutamine, and 1% penicillin/streptomycin. The E14 cell line of mESCs was cultured on precoated 0.2% gelatin (Sigma) tissue culture dishes in Glasgow Minimum Essential Medium, also containing 15% FBS (Biowest), Tylosine (Sigma), 0.1 mM β-mercaptoethanol (Gibco), supplemented with nonessential amino acids (Gibco), 1 mM sodium pyruvate (Gibco), LIF conditioned media, and GlutaMAX (Gibco). LIF-conditioned media was prepared as previously described^36^. All cell lines were grown at 37 °C in a 5% CO_2_ incubator and passaged every 2–3 days. For mESC differentiation, the cells were first washed with phosphate-buffered saline, then incubated in media without LIF, and harvested at the indicated time of differentiation. For EB differentiation, EBs were generated through trypsinization of mESC cultures and plating 1000 cells/drop using the hanging drop technique on a low attachment petri dish (Corning) in ESC medium lacking LIF. EBs were then transferred to a tissue culture dish after three days for further differentiation and harvested at the indicated time point.

### Plasmid vectors, siRNAs, and CRISPR

Full-length human or mouse TFEB, TFE3, Sox2, Oct4, or Nanog cDNA was cloned into pCMV4-Flag (Sigma) or pEGFP (Clontech) expression vectors. Point mutation constructs were made by PCR-based site-directed mutagenesis (SDM) method using KOD Plus Neo DNA polymerase (Toyobo). phOct4-eGFP was a gift from Wei Chu (Addgene plasmid #38776), mNanog5p-LUC reporter a gift from Austin Cooney (Addgene plasmid #16337), pGL3-*Sox2* promoter-Luc a gift from Yuh-Shan Jou (Addgene #101761), pOct4 promoter-Luciferase a gift from Shinya Yamanaka (Addgene #17221), and *TFEB* promoter-luciferase reporter a gift from Albert La Spada (Addgene #66801). For TFEB KO and TFEB/3 DKO generation, lentiCRISPRv2, a gift from Feng Zhang (Addgene #52961), was used^37^. The gRNA and si-RNAs sequences used in this study are shown in Supplementary Table 1.

### Transfections and infections

Cells were transfected using Lipofectamine 3000 (Invitrogen) or Lipofectamine RNAi-Max (Invitrogen) according to the manufacturer’s protocol. For virus production, HEK293T cells plated on 100 mm^2^ dishes were transfected with the indicated lentiviral transfer plasmid along with the packaging, and envelope plasmids. After 72 h, HEK293T medium containing the viral particles was collected, filtered through a 0.45-μm syringe filter (GVS Ref. FJ25ASCCA004FL01), and precipitated with PEG-it Virus Precipitation Solution (SBI System Biosciences Cat# LV810A-1). mESCs were then treated with viral stocks in presence of 10 μg/mL polybrene (Sigma). After two to three days of infection, cells were selected using the appropriate selection markers. ALP staining, cell morphology, and protein and mRNA levels were examined after infection and selection. ALP staining was performed using the ALP Kit (Milipore) according to the manufacturer’s protocol.

### Generation of TFEB KO and TFEB/3 DKO mESCs

The mESCs were seeded onto gelatin-coated 100-mm dishes in 15% FBS containing medium. Lentivirus stocks generated using the lentiviral vector CRISPRv2 were then added to a cell culture medium containing 10 μg/mL polybrene for 24 h. Two days later, the medium was then removed and a fresh culture medium containing 2.5 μg/mL puromycin (Sigma, no. P7255) was added and the cells were expanded in culture media containing 2.5 μg/mL puromycin. The cells were selected for two to three weeks after infection with all three CRISPRv2-gRNA TFEB virus stocks. Following 2 weeks of selection, colonies were picked and expanded in six-well plates. The clones were screened and characterized based on morphology and expression pattern of pluripotency markers. Similarly, for TFEB/3 DKO cell generation, mESCs were infected with both CRISPRv2-gRNA TFEB and TFE3 virus stocks and selected for two to three weeks before the clones were screened and characterized for morphology and expression pattern of pluripotency and differentiation markers.

### Luciferase reporter assay

For performing the luciferase reporter assay, cells in each 12-well plate were co-transfected with the indicated luciferase reporter plasmids for 24 h (pRL-TK Renilla gene as an internal control reporter and the indicated plasmids). After 24–48 h of transfection, cells were lysed and luciferase activities were analyzed and measured using a Dual-Luciferase reporter assay system using a GLOMAX 20/20 luminometer (#E1960; Promega).

### Western blotting and immunoprecipitation

Cell lysates were isolated using lysis buffer (20 mM Tris, pH 7.5, 100 mM NaCl, 1 mM EDTA, 2 mM EGTA, 50 mM β-glycerophosphate, 50 mM NaF, 1 mM sodium vanadate, 2 mM dithiothreitol, 1 mM phenylmethylsulfonyl fluoride (PMSF), 1 μg/mL leupeptin, and 1% Triton X-100) and allowed to lyse on ice for 30 min. Samples were then centrifuged at 14,000×*g* for 10 min at 4 °C, and their supernatant was collected for immunoprecipitation or immunoblotting. Protein concentration was determined through the Bradford assay. For immunoprecipitation, 600–800 μg of protein cell lysate was incubated with indicated antibodies as shown in the figures along with protein A/G plus agarose beads (Santa Cruz Biotechnology). Agarose beads were washed with wash buffer for 10 min and then centrifuged at 3000 × *g* for 3 min. The process was repeated five times. Equal amounts of protein were adjusted and boiled in Laemmli sample buffer and ran on 8% or 10% sodium dodecyl sulfate-polyacrylamide gel electrophoresis (SDS-PAGE) gels before being transferred to polyvinylidene fluoride membranes (Pall). These membranes were then probed with respective antibodies as indicated in the figures. Antibodies used were as follows: anti-Sox2 (#2748 CST), anti-TFEB (A303-673A Bethyl), anti-TFE3 (#ab93808 Abcam), anti-Nanog (#A300-397A Bethyl), anti-Oct4 (#ab19857 Abcam), anti-Flag (#F3165 Sigma), anti-GFP (sc-9996 Santa), anti-β-actin (A5441 Sigma), anti-vinculin (#4650S CST), anti-Tuj1 (#MAB1195 R&D), anti-Lamin B1 (sc-377000 Santa), anti-Phospho-TFEB (Ser211) (#37681 CST), anti-Phospho-TFEB (Ser142) (#ABE1791 Milipore Sigma), anti-β-tubulin (#GTX107175 GenTex) and anti-LC3B (#2775S CST).

### ChIP analysis

Cells were cross-linked with 1% formaldehyde (Sigma) on a shaker for 10 min at 25 °C and quenched with 0.125 M glycine for 10 min. Cells were washed with cold FBS prior to harvesting for nuclei isolation. Cell pellets were suspended in nuclei isolation buffer (50 mM HEPES-KOH pH 7.5, 140 mM NaCl, 1 mM EDTA, 10% Glycerol, 0.5% NP-40, 0.25% Triton X-100, and protease inhibitors), followed by centrifugation at 400 × *g* at 4 °C for 5 min to isolate the nuclei. The pellets were then lysed in ice-cold SDS lysis buffer (1% SDS, 10 mM EDTA. 50 mM Tris-HCl pH 8.0, 1 mM PMSF, 1 μg/mL leupeptin, and 1 μg/mL aprotinin). Chromatin was then sonicated twice for 10 cycles (30 s on and 30 s off). Sonicated samples were then centrifuged at 14,000 × *g* at 4 °C for 10 min. Dyna magnetic beads (Invitrogen) were precleared, prepared, and incubated overnight at 4 °C with the appropriate antibodies, according to the manufacturer’s protocol. Antibodies used for ChIP analysis were as follows: anti-IgG (Bethyl), anti-Sox2 (#2748 CST), anti-TFEB (#ab2636 Abcam), anti-TFE3 (#ab93808), anti-Nanog (#ab214549 Abcam), and anti-Oct4 (#ab19857 Abcam). Immunoprecipitation was performed by incubating antibody-bound beads with chromatin overnight at 4 °C. Beads bound to immunocomplexes were then washed with low salt (0.1% SDS, 1% Triton-X, 2 mM EDTA, 20 mM Tris-HCl pH 8.0, and 150 mM NaCl), high salt (0.1% SDS, 1% Triton X-100, 2 mM EDTA, 20 mM Tris-HCl pH 8.0, and 500 mM NaCl), and LiCl (250 mM LiCl, 1% NP-40, 1% deoxycholate, 1 mM EDTA, and 10 mM Tris-HCl pH 8.0) buffers sequentially for 10 min each and centrifuged. Washing was repeated once again with TE buffer (10 mM Tris-HCl pH 8.0 and 1 mM EDTA) that consisted of 50 mM NaCl and centrifuged at 960×*g* for 3 min at 4 °C. The supernatants were then removed and beads were suspended in elution buffer before incubation at 65 °C for 15 min. Next, centrifugation was performed at 16,000×*g* for 1 min, the supernatant was de-cross-linked by adding 200 mM NaCl, and incubated at 65 °C for overnight with shaking. DNA purification was performed by phenol–chloroform extraction, and purified DNA was used for qPCR analysis. Primers used for ChIP-qPCR are shown in Supplementary Table 2.

### Isolation of mRNA and qPCR analysis

RNA was isolated using TRIZOL reagent (Invitrogen) and according to the manufacturer’s protocol. cDNA was synthesized from 1 μg of RNA using ReverTra Ace qPCR RT kit (Toyobo). qPCR was carried out using diluted cDNA and appropriate primers through ABI prism 7000 Sequence Detector (Applied Biosystem) with Thunderbird SYBR Green qPCR Mix (Toyobo). Relative mRNA expression was calculated using the ΔΔCt method and each gene was normalized with Ct value of β-actin. All primers are listed in Supplementary Table 3.

### Lysosomal activity assay

Cells were labeled with Lysotracker Red DND-99 (Invitrogen) for 2 h at 1000× dilution or 20 μg/mL DQ Green BSA (Invitrogen) overnight in mESC medium before fixing with 4% paraformaldehyde for 20 min at 25 °C. Cells were then stained with Hoechst stain. To induce starvation, cells were cultured in serum free media for 16 h before harvesting. Images were acquired with fluorescence microscopy (Cell Observer, Zeiss).

### Cell fractionation assay

Cells were collected by centrifugation at 600 × *g* for 5 min at 4 °C. Cytosol extraction buffer and nuclear extraction buffer were prepared in advanced before adding to cell pellets. Nuclear and cytosol fractionation were performed using Nuclear/Cytosol Fractionation Kit (BioVision, cat#K266-25) according to the manufacturer’s protocol. Protein concentration was determined through the Bradford assay. Anti-Lamin B1 (sc-377000 Santa) and anti-β-tubulin (#GTX107175 GenTex) were used as nuclear and cytosol loading control respectively.

### Statistical analysis

All statistical data are expressed as the mean ± SD and number of sample size (*n* = 3) were indicated in each figure legends. Most of the experiments were repeated three times. The statistical significance of differences between different groups was analyzed using the Student’s *t* test. *P* values were calculated using Student’s *t* test and the values of *p < 0.05; **p < 0.01; ***p < 0.001 ****p < 0.0001 were considered significant.

## Supplementary information

Supplementary Tables

Supplementary Figure and Table Legends

Supplementary Figure 1

Supplementary Figure 2

Supplementary Figure 3

Supplementary Figure 4

Supplementary Figure 5

Supplementary Figure 6

Supplementary Figure 7

Supplementary Figure 8
