# Supplementary material for: TFEB regulates pluripotency transcriptional network in mouse embryonic stem cells independent of autophagy–lysosomal biogenesis
Source: Cell Death Dis. 2021 Apr 1;12(4):343. doi: 10.1038/s41419-021-03632-9 (PMC8016867; doi:10.1038/s41419-021-03632-9)
Supplement: Supplementary file 1 — Supplementary Tables [file 41419_2021_3632_MOESM1_ESM.pdf]

Supplementary Tables for

TFEB Regulates Pluripotency Transcriptional Network in Mouse Embryonic Stem Cells Independent of Autophagy-lysosomal Biogenesis

Anderson Tan[†], Renuka Prasad[†] and Eek-hoon Jho[1]

Department of Life science, University of Seoul, Seoul, Republic of Korea, 130-743, Republic of Korea.

[†] These authors contributed equally to this work.

[1] To whom correspondence may be addressed. E-mail: ej70@uos.ac.kr

Tel: +82-02-6490-2671, Fax: +82-02-6490-2664.

Supplementary Table 1. Sequences for siRNA and gRNA

**gRNA sequences for lentiCRISPRv2**	
TFEB gRNA-1	CACCGCATATTCACACCCGACGGCG
TFEB gRNA-2	CACCGCAGCCCGATGCGTGACGCCA
TFEB gRNA-3	CACCGTCGGGCTCCCTGTAGTCGAG
TFE3 gRNA-1	CACCGACAACGTTCCATGTATCGGG
TFE3 gRNA-2	CACCGGGCGATTCAACATTAACGAT
TFE3 gRNA-3	CACCGAGCGTGTAGGGTTCTCGAGG
**siRNA sequences**	
si-GFP sense	GTTCAGCGTGTCCGGCGAG
si-GFP antisense	CTCGCCGGACACGCTGAAC
si-Oct4 sense	GCCGACAACAATGAGAACCTT
si-Oct4 antisense	AAGGTTCTCATTGTTGTCGGC
si-Sox2 sense	GAAGGAGCACCCGGATTA
si-Sox2 antisense	TAATCCGGGTGCTCCTTC
si-Nanog sense	GTTAAGACCTGGTTTCAAA
si-Nanog antisense	TTTGAAACCAGGTCTTAAC
si-TFE3 sense	GCCTAACATCAAACGCGAGAT
si-TFE3 antisense	ATCTCGCGTTTGATGTTAGGC
si-ATG7 sense	CTCAAGCTGATGTGCTGGCTT
si-ATG7 antisense	GCCAGCACATCAGCUTGAGTT

Supplementary Table 2. Sequences for ChIP-qPCR

**Primers used for ChIP-qPCR**	
**Nanog promoter**	
T1-F	AGAGAGAGAGAGAGAGAGTGGTGTAAA
T1-R	ATAATTAAACCAGCCATTTCCTTTTTTGT
T2-F	GCAGTGGAGACAGTAGTAGTATGGT
T2-R	GTTATTATTCTAGGCAGGGTTTCTCTG
T3-F	GCTTAGTCTTTTGTAGAATAAAACCCAG
T3-R	CTTCCTATTTCCACATCCCAAGTTCAAA
T4-F	GAGGATGCCCCCTAAGCTTTCCCTCCC
T4-R	CCTCCTACCCTACCCACCCCCTATTCTCCC
Oct4-F	GAGGATGCCCCCTAAGCTTTCCCTCCC
Oct4-R	CCTCCTACCCTACCCACCCCCTATTCTCCC
**Sox2 promoter**	
F	CGGTGAGAAGAGGGGGTGAGTGGGTGCTGGCGACAAGG
R	GGATACATAAGGGTGGATGGGGCGCGGGAAGCTGGGGC
**TFEB promoter**	
Sox2-F	CCAGTCTCATGTACTCAGACTGCCAGGCACTG
Sox2-R	CGTATCTGAGACCATGACAGTACAGTTAGGGAC
Oct4-F	CCCAACGGCTAGAGGCCGAAGGTCTAGCAGTAAACAAACAG
Oct4-R	ACCTCCTCCGAGAGACTCTAATGTCACCTCAGAGTGCCATTTCAGGA CCACTGTGC

Nanog-F	CCCAACGGCTAGAGGCCGAAGGTCTAGCAGTAAACAAACAG
Nanog-R	ACCTCCTCCGAGAGACTCTAATGTCACCTCAGAGTGCCATTTCAGGA CCACTGTGC
**Lysosomal promoter**	
CLCN7-F	GGATTTTGAGTTCTAGGTTTCTACCCTGGGCTGCAACGACC
CLCN7-R	CAAGACACTTTCTTAGAGACGTTGGCCATGGCCCACTGAG
CTSA-F	GGGAACCCTTCTGTCAACACCAAGATCACCTGATCTCTGG
CTSA-R	CCGTACCTCTGCTTCCTTGCGTCCTTGCTCTCCAGGA
CTSD-F	GACCACATCCTGGTAGTGGGCACCCCAATTATGCAAAAA
CTSD-R	ACTCCAGGCCTACGCACGGCTCTCGGGAACG
LAMP1-F	TCACGTGCGGAACGGAGCCTCCCGAAGAAGTGAGGAG
LAMP1-R	TTGCGCGGGCGGCGAGGCCGGCGGGAGGG
MCOLN1-F	CCTTTTGTTAGAGTTGCTATCGGCACTAGGACCGCGCG
MCOLN1-R	TCACTGCAGCCTCGCTCCCTCCCGGCGCCGCTTCAAA
TPP1-F	GCTCAACACAGCATAGAATTCTAATAGCAGCCCCAGCATTCAG
TPP1-R	ATGTAATCCCTCTAATGTAATCCCCCTTAGCTACCCTCCCCA

Supplementary Table 3. Primers for qPCR

qPCR primers	
mATG7-F	GTTCGCCCCCTTTAATAGTGC
mATG7-R	TGAACTCCAACGTCAAGCGG
mUVRAG -F	GCAGACCACGAGACAGTTGA
mUVRAG -R	CATCGTGACGTTGCACACAG
mWIPI1-F	CACAGGATGGAGGAGAATGTG
mWIPI1-R	TGCAGCATAAGATGGAGGTAAG
mATG9b -F	AGCTATCATCAGCGGAATGG
mATG9b -R	GCGAAGGAGGAAGGTTGTAA
SOX2-F	CATGAGAGCAAGTACTGGCAAG
SOX2 -R	CCAACGATATCAACCTGCATGG
OCT4-F	CCAATCAGCTTGGGCTAGAG
OCT4-R	CCTGGGAAAGGTGTCCTGTA
NANOG-F	TCTTCCTGGTCCCCACAGTTT
NANOG-R	GCAAGAATAGTTCTCGGGATGAA
mTFEB-F	CGGACAGATTGACCTTCAGAG
mTFEB-R	GCTGCTGCTGTTGCATATAAT
mTFE3-F	GGAAACAGAGAAGTCTCCCTTAAA

mTFE3-R	CTGAGTGGGTGGGTTCAATAA
MITF-F	AGGACCTTGAAAACCGACAG
MITF-R	GGTGGATGGGATAAGGGAAAG
TFEC-F	AGTGATACAATATGCCCCTTTGG
TFEC-R	GTTGATGTCTTGAGCCTGTTTG
SOX17-F	ATACGCCAGTGACGACCAGAG
SOX17-R	ACCACCTCGCCTTTCACCTTT A
OTX2-F	GCGAAGGGAGAGGACGACTTT
OTX2-R	CTGCTGTTGGCGGCACTTAG
NEURO D1-F	TATTGCGTTGCCTTAGCACT
NEURO D1-F	CATCCTCTTGAGTGTTATGG
GATA4-F	GAGGGTGAGCCTGTATGTAATG
GATA4-R	CTAGTGGCATTGCTGGAGTTA
HAND1-F	CACCACCTACCACCGCAGTA
HAND1-R	CCTTCTTGGGTCCTGAGCCTTT
BRACHYURY-F	TCTCTGGTCTGTGAGCAATGGT
BRACHYURY-R	TGCGTCAGTGGTGTGTAATGTG
mβ-actin-F	ATGGAGCCACCGATCCACA
mβ-actin-R	CCGTAAAGACCTCTATGCCAAC

mATG4b-F	TATGATACTCTCCGGTTTGCTGA
mATG4b-R	GTTCCCCCAATAGCTGGAAAG
mLAMP1-F	CAGCACTCTTTGAGGTGAAAAAC
mLAMP1-R	ACGATCTGAGAACCATTCGCA
mATP6v0E1-F	GCATACCACGGCCTTACTGT-
mATP6v0E1-R	GAGGATTGAGCTGTGCCAGA
mATP6v1h-F	ACTCCCCGAGGCTATCCAG
mATP6v1h-R	CACGAACTTCAGCAGCCTTG
mCSTD-F	TACTCCATGCAGTCATCGCC
mCSTD-R	GACGACTGTGAAACACTGCG
mMCOLN1-F	TCATTGCACTCATCACCGGC
mMCOLN1-R	CCAGATGTGGGGCTATCCTG
mCLCN7-F	CACGGCCAGGGAAGTAATGAG
mCLCN7-R	CGCAGGATCAAGCCTTGGAG
mCTSA-F	CAGCCCCTTCCAACTACCTC
mCTSA-R	CCGTTGTAGAGCAGGATCTGG
hVSP11-F	AAGAACCTCATGCCACCTCTTC
hVSP11-R	GGTAGTCCCTGATGACGGAGA
hCTSF-F	ACAGAGGAGGAGTTCCGCACTA

hCTSF-R	GCTTGCTTCATCTTGTTGCCA
hLAMP1-F	ACGTTACAGCGTCCAGCTCAT
hLAMP1-R	TCTTTGGAGCTCGCATTGG
hATPv0E1-F	CATTGTGATGAGCGTGTTCTGG
hATPv0E1-R	AACTCCCCGGTTAGGACCCTTA
hLAMP2-F	GCCGTTCTCACACTGCTCTA
hLAMP2-R	TGAAATGCTCCAGACACTGAA
hTFEB-F	CCAGAAGCGAGAGCTCACAGAT
hTFEB-R	TGTGATTGTCTTTCTTCTGCCG
hUVRAG-F	CTGTTTGGATGGGCTGAAAT
hUVRAG-R	TGCGAACACAGTTCTGATCC
hATG9b-F	TGTGAGCCAGAAAAACAAGCAAA
hATG9b-R	GGTATGGGGGTGGGGTCTAA
hβ-actin-F	GAGATCACTGCCCTGGCACC
hβ-actin-R	GATGGAGGGGCCGGACTCG
hUVRAG -F	CTGTTTGGATGGGCTGAAAT
hUVRAG -R	TGCGAACACAGTTCTGATCC
