# Supplementary material for: TFEB regulates pluripotency transcriptional network in mouse embryonic stem cells independent of autophagy–lysosomal biogenesis
Source: Cell Death Dis. 2021 Apr 1;12(4):343. doi: 10.1038/s41419-021-03632-9 (PMC8016867; doi:10.1038/s41419-021-03632-9)
Supplement: Supplementary file 2 — Supplementary Figure and Table Legends [file 41419_2021_3632_MOESM2_ESM.docx]

**Supplementary Figure and Table Legends for**

**TFEB Regulates Pluripotency Transcriptional Network in**

**Mouse Embryonic Stem Cells Independent of**

**Autophagy-lysosomal Biogenesis**

Anderson Tan^†^, Renuka Prasad^†^ and Eek-hoon Jho^1^

Department of Life science, University of Seoul, Seoul, Republic of Korea, 130-743, Republic of Korea.

^†^ These authors contributed equally to this work.

^1^ To whom correspondence may be addressed. E-mail: [ej70@uos.ac.kr](mailto:ej70@uos.ac.kr)

Tel: +82-02-6490-2671, Fax: +82-02-6490-2664.

**Supplementary Figure Legends**

**Supplementary Figure 1, related to Figure 1:** **Dynamic changes of TFEB levels during mESC differentiation.**

**(a)** Endogenous TFEB protein levels in mESCs from Day 0 to Day 9 in LIF withdrawal condition. **(b)** qPCR for TFEB and Oct4 mRNA levels in mESCs from Day 0 to Day 9 in LIF withdrawal condition. **(c)** Endogenous TFEB protein levels during differentiation after forming EB. **(d)** qPCR for TFEB and Oct4 mRNA levels in mESCs during differentiation after forming EB. **(e,f)** qPCR for mRNA expression of differentiation markers from three germ layers; mesoderm (GSC), endoderm (GATA4) and ectoderm (FGF5) during LIF withdrawal differentiation **(e)** and differentiation by forming EB **(f).** **(g)** mRNA levels of MiT family members (TFEB, TFE3, MITF and TFEC) from Day 0 to Day 9 in LIF withdrawal induced differentiation. Day 5 (D5) TFEB sample was used as control for all groups. Sox17 was used as a positive control for differentiation. mRNA was normalized with β-actin. All statistical analyses represent average values of a representative experiment from at least 2 independent experiments. Error bars represent SD values of triplicate assays. Data are shown as mean ± SD, n=3. *, p <0.05; **, p <0.01; ***, p <0.001 ****, p <0.0001 compared to the corresponding control group. Student’s t test was used for all statistical analysis.

**Supplementary Figure 2, related to Figure 1: Autophagy/lysosomal inhibition does not affect mESC pluripotency.**

**(a)** Morphology picture of si-RNA mediated ATG7 reduction and Bafilomycin treated (100 nM) mESC as compared to control. Scale bars, 100 μm. **(b)** Western blot analysis of stem cell markers and LC3 levels in ATG7-knockdown and Bafilomycin treated mESCs.

**Supplementary Figure 3, related to Figure 2: TFE3 does not regulate the expression of Nanog.**

**(a)** Ectopic expression of active TFEB but not TFE3 induces Nanog promoter-driven luciferase activity in HEK293T cells. **(b)** ChIP-qPCR analysis was performed by pulling-down with anti-TFE3 or anti-TFEB antibody, following which qPCR targeting the T4 site of Nanog promoter at the genomic DNA level in mESCs was performed. **(c)** TFE3 knockdown by si-RNA does not affect expression of *Nanog* and *Sox2*. mRNA was normalized with β-actin. All statistical analyses represent average values of a representative experiment from at least 2 independent experiments. Error bars represent SD values of triplicate assays. Data are shown as mean ± SD, n=3. *, p <0.05; **, p <0.01; ***, p <0.001 ****, p <0.0001 compared to the corresponding control group. Student’s t test was used for all statistical analysis.

**Supplementary Figure 4, related to Figure 3: TFEB does not regulate the expression of Oct4.**

**(a)** Ectopic expression of active TFEB (TFEB-AA) does not induce Oct3/4 promoter-driven luciferase activity in HEK293T cells. **(b)** No Oct3/4 promoter-driven GFP signal (OCT3/4p-GFP) was detected when mCherry-TFEB was overexpressed in HEK293T cells. Scale bars, 100 μm. **(c)** No interaction was observed between TFEB and Oct3/4 at the endogenous level. Immunoprecipitates from lysates of mESCs with anti-IgG or TFEB antibody were immunoblotted with anti-TFEB antibody. All statistical analyses represent average values of a representative experiment from at least 2 independent experiments. Error bars represent SD values of triplicate assays. Data are shown as mean ± SD, n=3. *, p <0.05; **, p <0.01; ***, p <0.001 ****, p <0.0001 compared to the corresponding control group. Student’s t test was used for all statistical analysis.

**Supplementary Figure 5, related to Figure 4: Analysis of endogenous LC3, lysotracker and DQ-BSA staining of mESC in nutrient, starvation and differentiation conditions.**

**(a**) Lysotracker staining of D0, D5 embryonic body (EB) and D9 EB. Nuclei were labeled with Hoechst staining. Scale bars, 100 μm. **(b)** Increase of autophagy flux during mESC LIF withdrawal differentiation (mESC, D5 and D9). LC3 is an autophagy marker. (**c, d**) mESCs were starved for 24 h prior to labeling with either Lysotracker Red (**c**) or DQ-BSA (**d**). Nuclei were labeled with Hoechst staining (blue). Scale bars, 100 μm**.**  **(e)** mESCs were serum-starved for 24 h and levels of TFEB and stem cell markers were examined.

**Supplementary Figure 6, related to Figure 4: Regulation of genes involved in autophagy-lysosomal biogenesis by TFEB in HEK293 cells and differentiated cells.** (**a**) GFP-TFEB-S142A was overexpressed in HEK293 cells for 48 h prior to labeling with Lysotracker Red. Nuclei were stained with Hoechst. Scale bars, 100 μm. (**b,c**) qPCR analysis to measure expression of genes involved in lysosomal biogenesis (**b**) and autophagy (**c**) by ectopic expression of GFP-TFEB-AA in HEK293 cells. mRNA was normalized with β-actin. All statistical analyses represent average values of a representative experiment from at least 2 independent experiments. Error bars represent SD values of triplicate assays. Data are shown as mean ± SD, n=3. *, p <0.05; **, p <0.01; ***, p <0.001 ****, p <0.0001 compared to the corresponding control group. Student’s t test was used for all statistical analysis.

**Supplementary Figure 7, related to Figure 4: Increased of autophagy/lysosomal functions in TFEB KO mESC is mediated by TFE3.**

(**a**) GFP-TFE3 was overexpressed in mESCs for 24 h prior to harvest. qPCR analysis to measure expression of genes involved in lysosomal biogenesis. (**b**) ChIP-qPCR analysis was performed by pulling-down with anti-TFE3 antibody, following which qPCR targeting the lysosomal promoters at the genomic DNA level in mESCs was performed. (**c**) qPCR analysis of expression of genes involved in autophagy-lysosomal biogenesis in WT, TFEB KO and TFEB/3 DKO cells. mRNA was normalized with β-actin. (**d**) WT, TFEB KO and TFEB/3 DKO cells were labelled with Lysotracker Red for 2 hours. Nuclei were stained with Hoechst. Scale bars, 100 μm. **(e)** Increased autophagy flux in TFEB KO was abrogated in TFEB/3 DKO cells. **(f)** Autophagy flux was determined in WT and TFEB KO mESC in both undifferentiated and differentiated state (D9) upon Bafilomycin A1 treatment (50nM) for 12 hours. All statistical analyses represent average values of a representative experiment from at least 2 independent experiments. Error bars represent SD values of triplicate assays. Data are shown as mean ± SD, n=3. *, p <0.05; **, p <0.01; ***, p <0.001 ****, p <0.0001 compared to the corresponding control group. Student’s t test was used for all statistical analysis.

**Supplementary Figure 8, related to Figure 5: Reduction of TFEB promoter-driven luciferase activity upon mutation of Sox2, Oct4 or Nanog putative binding sites.**

(**a-c**) Mutation in putative Sox2, Oct4 or Nanog binding sites on TFEB promoter reduces the TFEB promoter-driven luciferase activity in HEK293T cells. HEK293T cells were co-transfected with luciferase reporter constructs and Flag-Sox2, Oct4, or Nanog, respectively, as depicted. Luciferase activity was evaluated 24 h after transfection. All statistical analyses represent average values of a representative experiment from at least 2 independent experiments. Error bars represent SD values of triplicate assays. Data are shown as mean ± SD, n=3. *, p <0.05; **, p <0.01; ***, p <0.001 ****, p <0.0001 compared to the corresponding control group. Student’s t test was used for all statistical analysis.

**Supplementary Table Legends**

**Supplementary Table 1. Sequences for siRNAs and gRNAs.**

Supplementary table 1 shows the complete siRNA and gRNA sequences used in this study.

**Supplementary Table 2. Sequences for ChiP-qPCR**

Supplementary table 2 shows the complete primer sequences used for ChIP-qPCR. ChIP-qPCR for Nanog promoter, Sox2 promoter, TFEB promoter and Lysosomal promoter were performed using primers listed in the table.

**Supplementary Table 3. Primers for qPCR**

Supplementary table 3 shows the primers for real time qPCR used in this study.
