# Supplementary figures and images for: TFEB regulates pluripotency transcriptional network in mouse embryonic stem cells independent of autophagy–lysosomal biogenesis
Source: Cell Death Dis. 2021 Apr 1;12(4):343. doi: 10.1038/s41419-021-03632-9 (PMC8016867; doi:10.1038/s41419-021-03632-9)

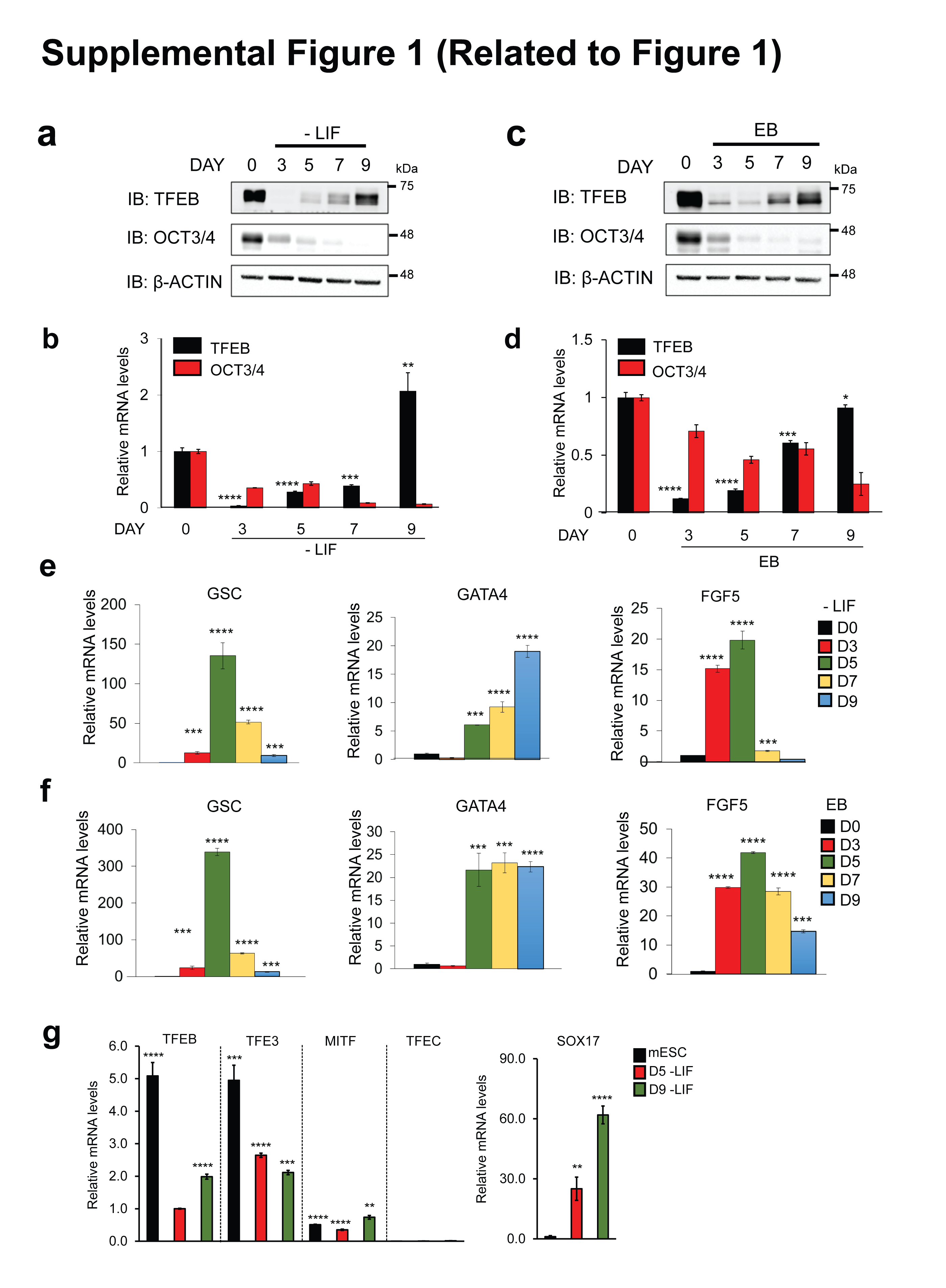

Supplement: Supplementary file 3 — Supplementary Figure 1 [file 41419_2021_3632_MOESM3_ESM.tif]

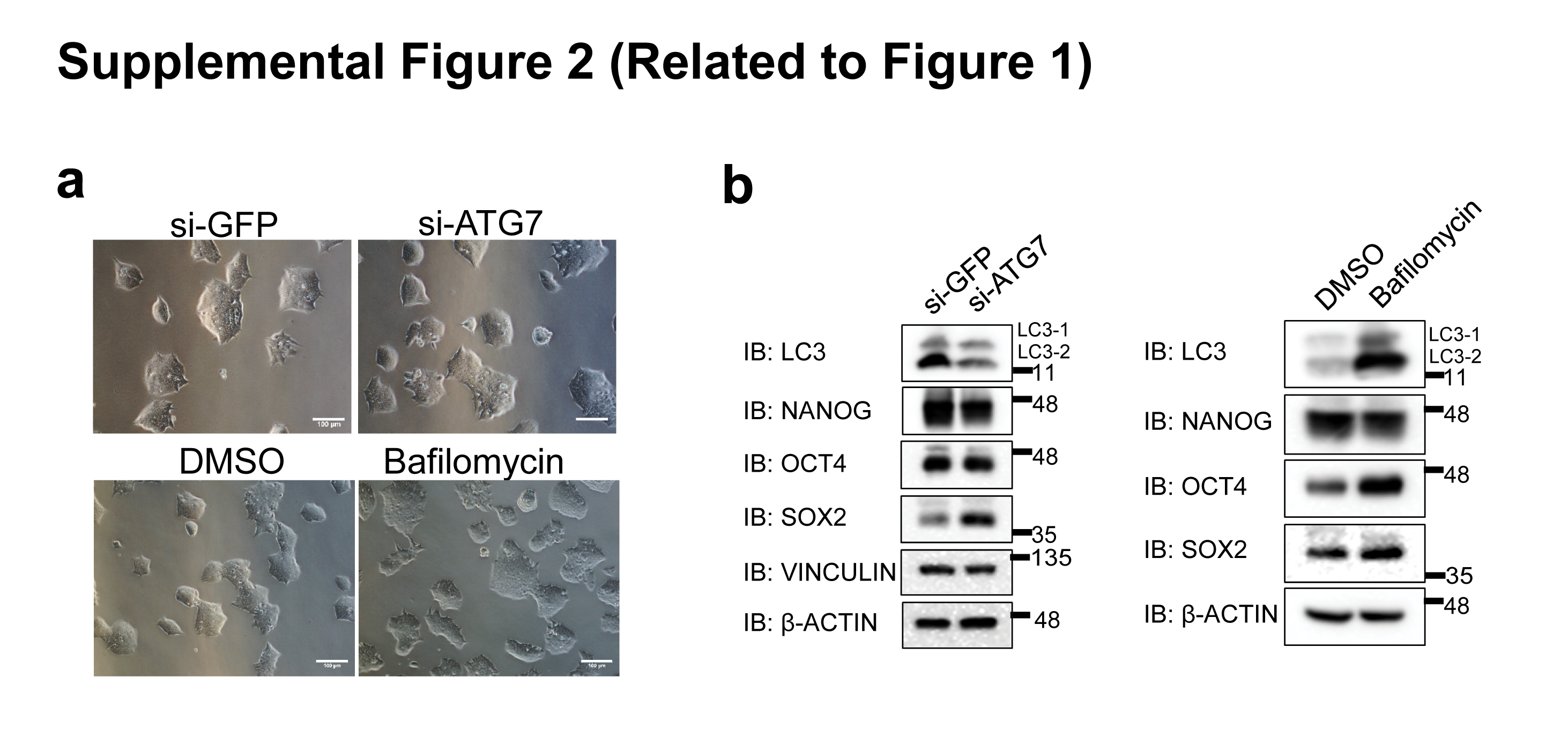

Supplement: Supplementary file 4 — Supplementary Figure 2 [file 41419_2021_3632_MOESM4_ESM.tif]

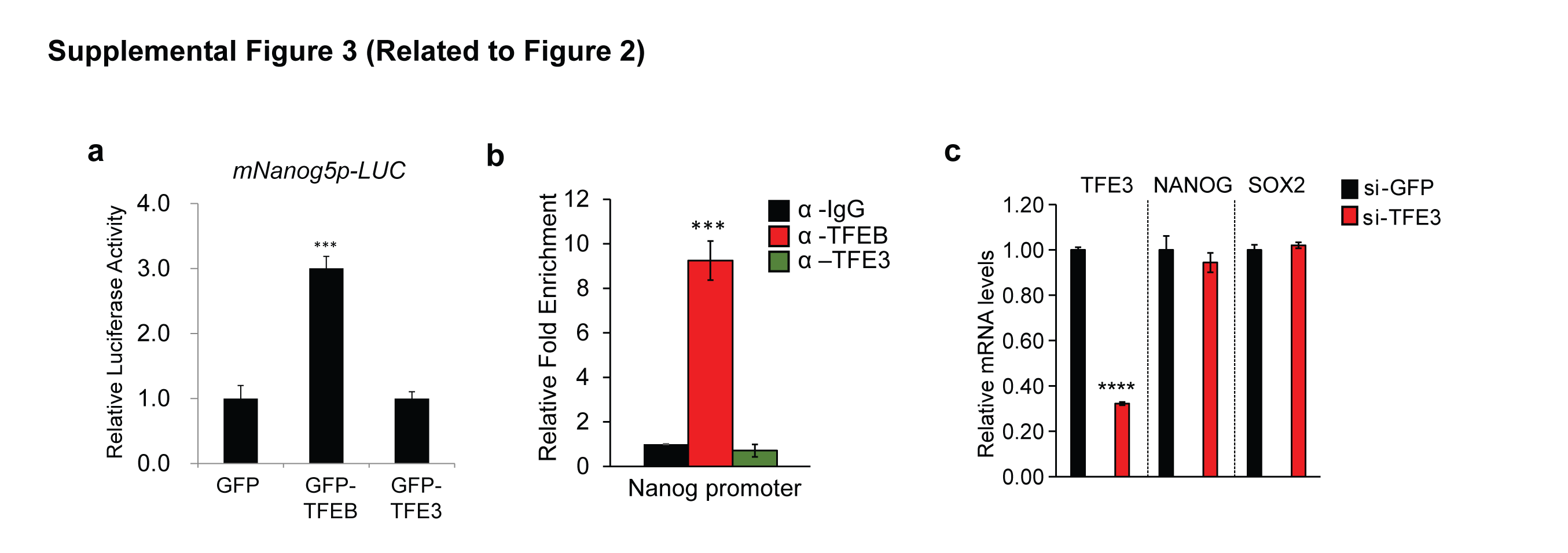

Supplement: Supplementary file 5 — Supplementary Figure 3 [file 41419_2021_3632_MOESM5_ESM.tif]

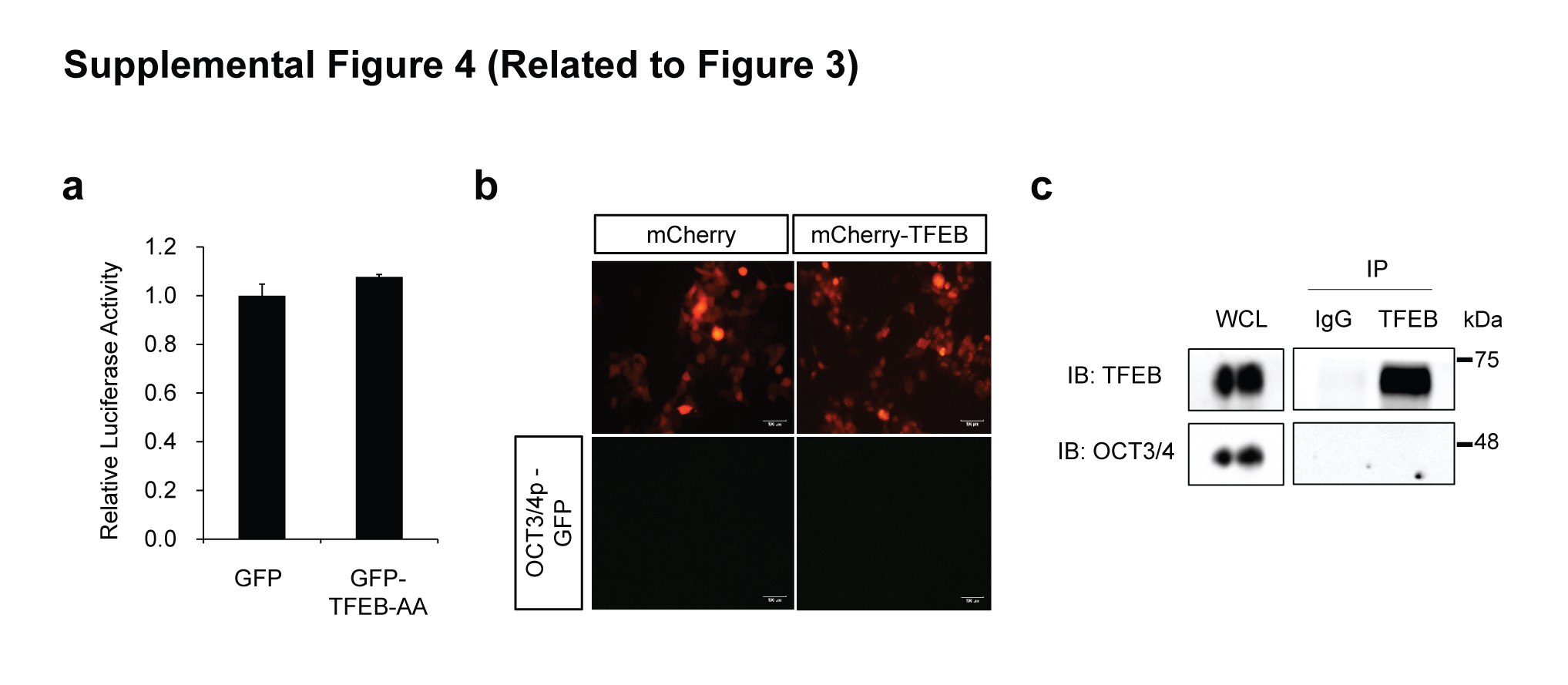

Supplement: Supplementary file 6 — Supplementary Figure 4 [file 41419_2021_3632_MOESM6_ESM.tif]

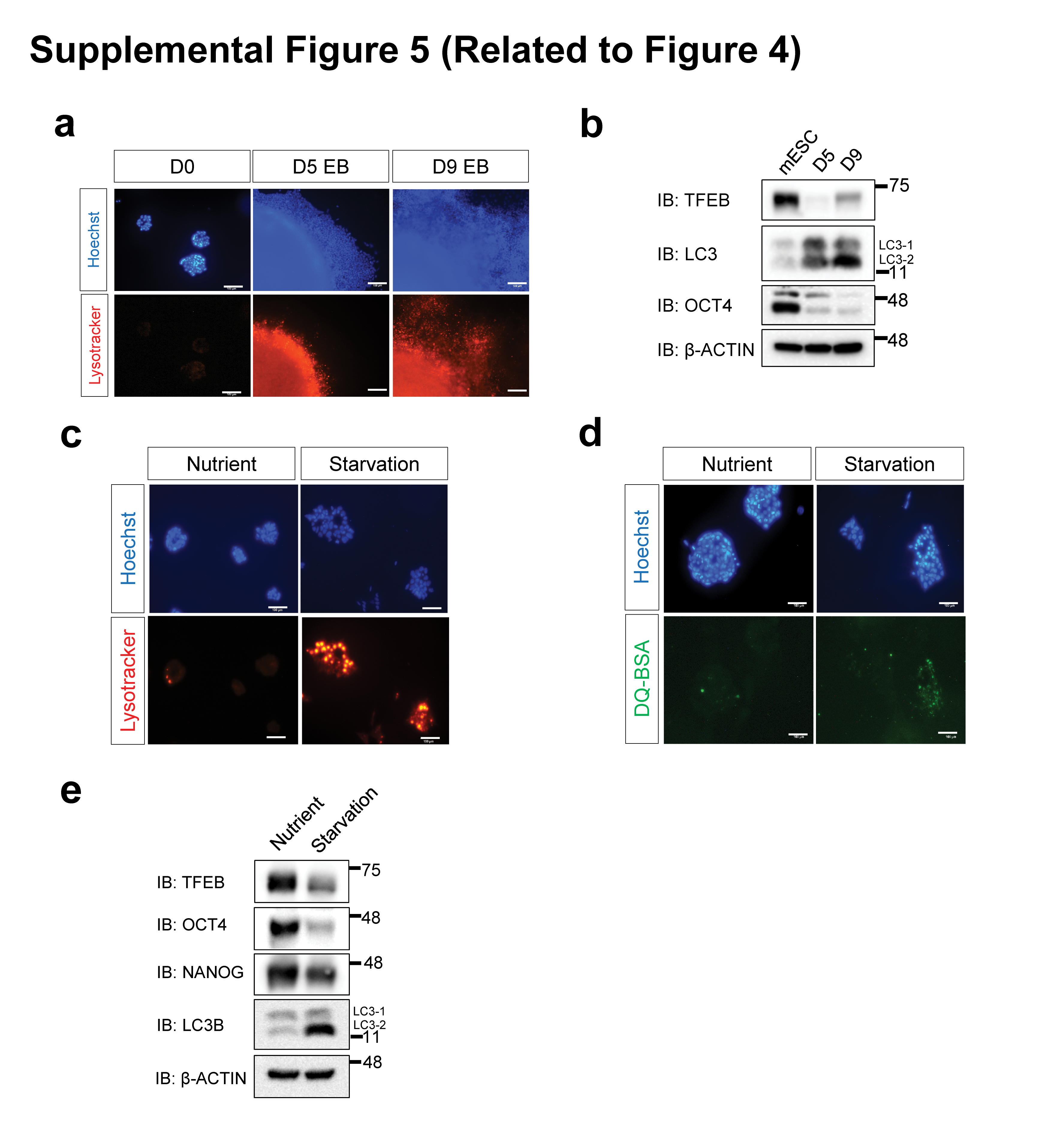

Supplement: Supplementary file 7 — Supplementary Figure 5 [file 41419_2021_3632_MOESM7_ESM.tif]

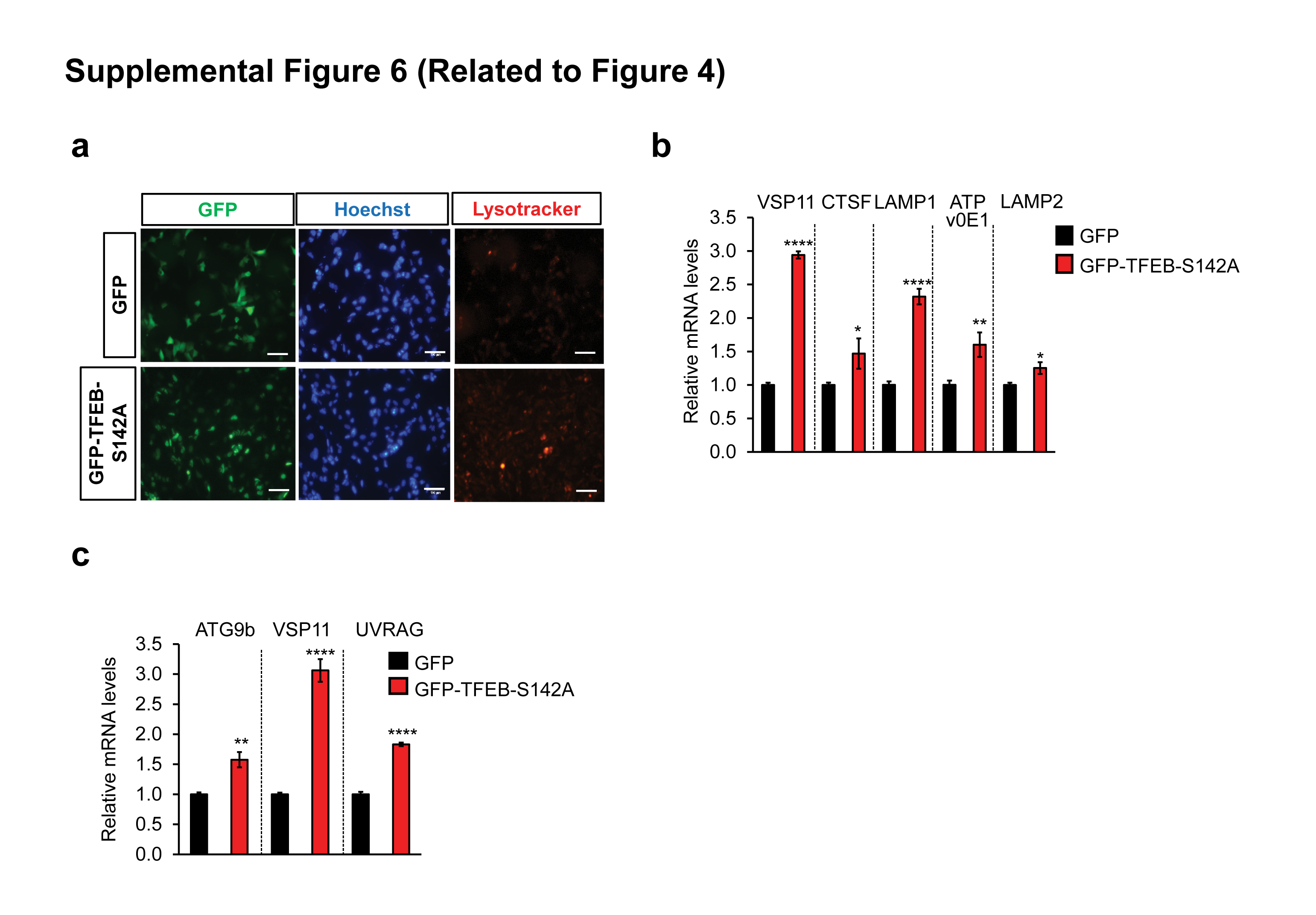

Supplement: Supplementary file 8 — Supplementary Figure 6 [file 41419_2021_3632_MOESM8_ESM.tif]

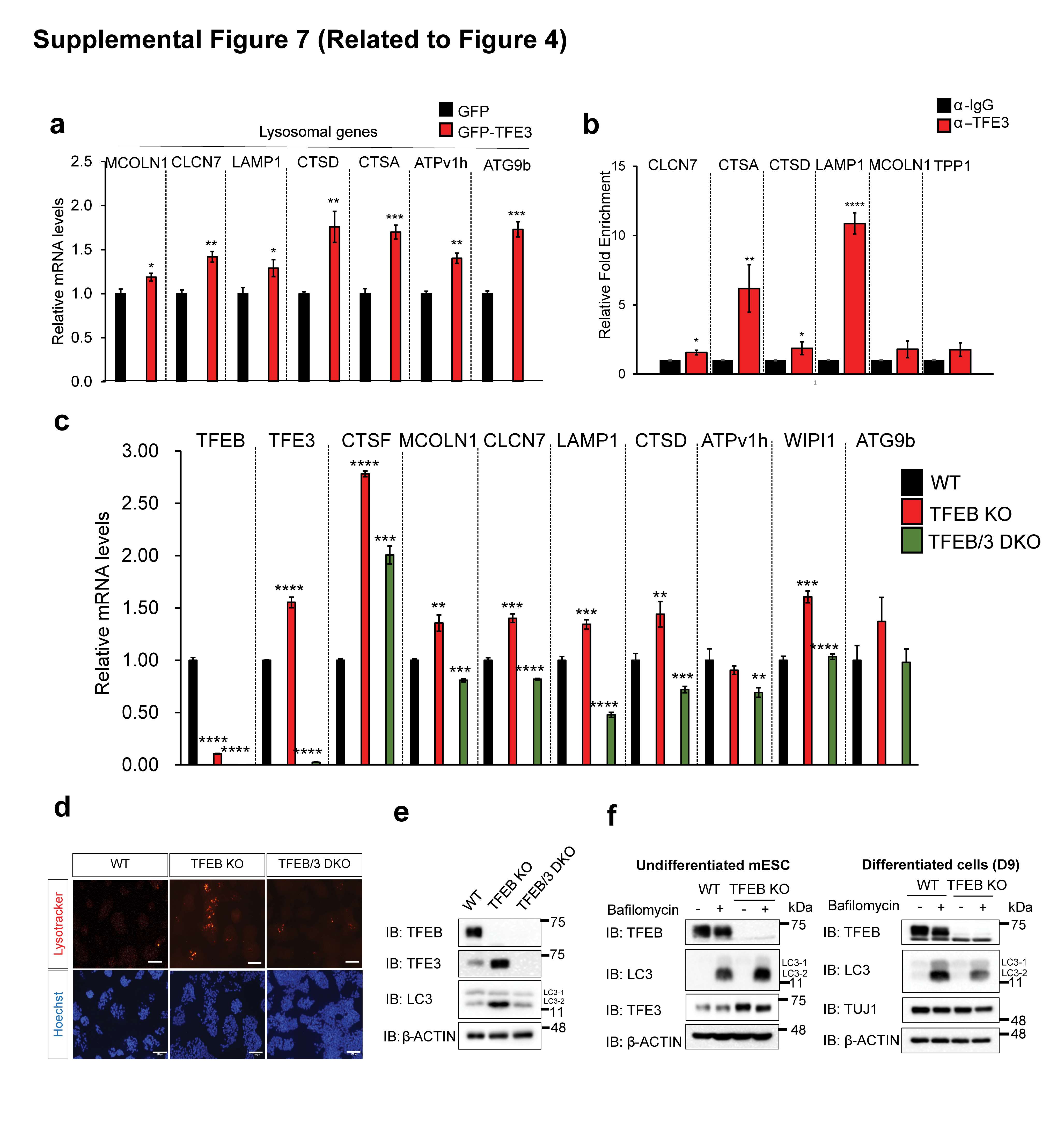

Supplement: Supplementary file 9 — Supplementary Figure 7 [file 41419_2021_3632_MOESM9_ESM.tif]

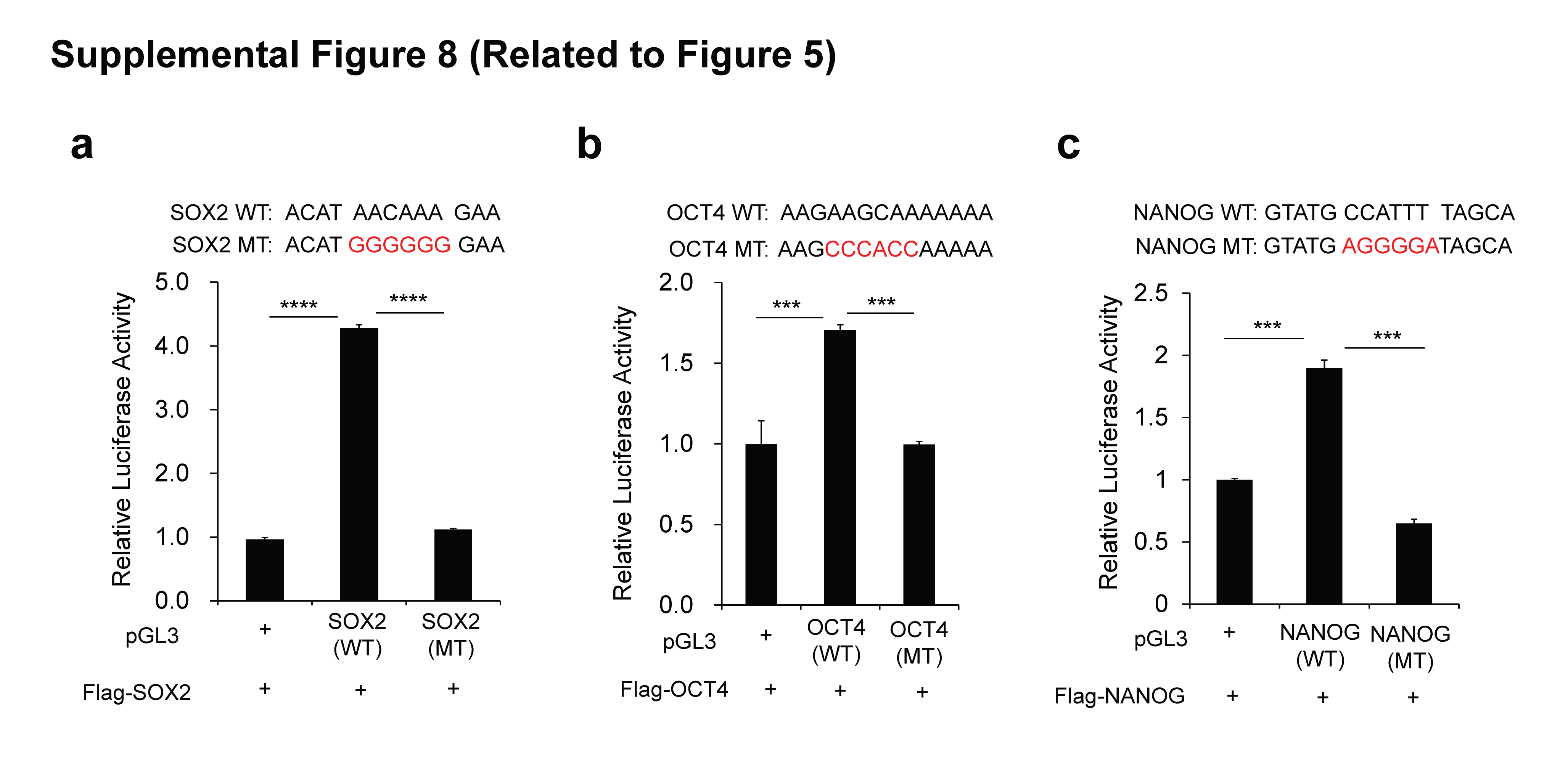

Supplement: Supplementary file 10 — Supplementary Figure 8 [file 41419_2021_3632_MOESM10_ESM.tif]
